# Plant-Based Meat Alternatives on the Island of Ireland: Changes in the Market and Comparisons with Conventional Meat

**DOI:** 10.3390/foods14050903

**Published:** 2025-03-06

**Authors:** Leona Lindberg, Jayne V. Woodside, Hannah Fitzgerald, Niamh Campbell, Hannah Vogan, Ciara Kelly, Mehrnoush Robinson, Anne P. Nugent

**Affiliations:** 1 Centre for Public Health, School of Medicine, Dentistry and Biomedical Sciences, Queen’s University Belfast, Belfast BT12 6BJ, UK; j.woodside@qub.ac.uk; 2 Institute for Global Food Security, School of Biological Sciences, Queen’s University Belfast, Belfast BT9 5DL, UKa.nugent@qub.ac.uk (A.P.N.); 3 UCD Institute of Food and Health, School of Agriculture and Food Science, University College Dublin, Belfield, D04 V1W8 Dublin, Ireland

**Keywords:** plant-based meat alternatives, meat products, A-scores, packaging, allergens, ingredients, nutritional quality

## Abstract

The plant-based meat alternative (PBMA) market and consumer base on the island of Ireland (IOI) has grown rapidly in recent years. Therefore, this study compared the changes in PBMAs available on the IOI over time and the nutritional profiles of PBMAs with meat counterparts. Three online audits of PBMAs available in Tesco’s and Sainsbury’s in 2021/2022, 2022/2023 and 2023/2024 were conducted. All on-pack information was extracted and analysed using descriptive statistics to identify trends over time. The nutritional content of selected categories of PBMAs identified in the 2023/2024 audit was compared to similar categories of meat using independent samples *t*-tests or Mann–Whitney-U tests. The number of PBMAs available on the IOI has increased over time (n = 350, n = 321 and n = 398), with a trend in slight improvements in A-scores observed for most categories. Compared to meat, PBMAs had significantly lower total and saturated fat contents and higher carbohydrate, fibre and sugar contents across most categories. The increase in PBMAs over time suggests that the development of new products is ongoing. However, the higher salt contents of PBMAs compared to meat in some categories and only slight improvements in A-scores suggest that focus should be redirected to product reformulation and refinement to improve the nutritional quality of PBMAs.

## 1. Introduction

Recommendations to reduce meat intake and increase consumption of plant-based foods have stemmed from the growing body of evidence linking dietary patterns high in meat, particularly red and processed meat, with increased risk of negative health outcomes [1,2,3,4] and a greater environmental impact [5,6]. The food industry has responded to the rise in consumer concerns over animal welfare and the environmental and health impact of meat production and consumption by developing and offering plant-based meat alternatives (PBMAs) [7]. PBMAs are products made from mostly plant-based ingredients, such as soy, pea and wheat protein, to replicate the taste, texture, appearance and functionality of meat [8,9,10].

Investment, innovation and sales in the PBMA market have grown rapidly in recent years with further growth predicted [11,12]. According to research by Barclays (2019), the PBMA share of the global meat market is expected to reach 10% by 2029, a ten-fold increase compared to 2019 figures [12]. Investment has fuelled innovation in the market, with the number of newly launched PBMAs in 2015–2019 exceeding 4400 products worldwide [13]. The UK is considered the most developed market within Europe, accounting for a third of all EU sales [11]. UK and EU consumers have responded to the increased visibility, accessibility and variety of PBMAs, with sales within the UK and EU more than doubling over the last decade [11]. Over the same period, reported increases in plant-based alternative consumption have been captured in national diet surveys, including the UK National Diet and Nutrition Survey [14] and Ireland’s National Adult Nutrition Survey [15]. Alongside a growth in the volume of products sold and consumed, a growth in the consumer-base for PBMAs in the UK and Ireland has also been reported, with 65% of the UK population reporting consumption of PBMAs in 2019 compared to only 50% in 2017 [16].

A number of audit and cross-sectional studies have assessed the nutritional content of PBMAs available in different markets [13,17,18,19,20,21,22,23,24,25,26,27,28,29,30,31,32,33,34,35,36,37,38,39], with these studies reporting a great degree of variability in the nutrient content of PBMAs within and between product categories. Whilst some inconsistencies exist in the literature, in general, when compared to meat equivalent products, PBMAs have been found to contain less total and saturated fat and higher carbohydrate and fibre content per 100 g, with protein and salt content dependent on the category of products assessed [13,18,19,21,22,24,25,27,32,34,35,36,39,40,41]. Fortification of PBMAs with micronutrients typically found in animal meat such as iron and vitamin B12, has been found to vary considerably across different countries [35]. A recent cross-sectional analysis by ProVeg International compared the nutritional profiles of different categories of meat products and comparable PBMAs (n = 422) available across 11 different countries and 4 continents [35]. Using a scoring system guided by a combination of the WHO European Nutrient Profile Model (NPM), the Netherlands Nutrition Centre White Paper and the European Food Safety Agency (EFSA) nutrition claim legislation, the analysis found that, overall, PBMAs have a slightly better nutritional score than meat products [35]. Variation in nutritional scores for PBMAs was observed across countries and categories, with PBMAs available in the Netherlands scoring the best, and PBMAs available in Malaysia scoring the worst [35]. Therefore, highlighting the need for country-specific data when assessing PBMAs in different contexts. PBMAs in the ‘Bacon,’ ‘Chicken nuggets’ and ‘Sausages’ categories had better nutritional scores on average than meat counterparts, whilst PBMAs in the ‘Schnitzel’ category had poorer nutritional scores than meat-based schnitzel [35], emphasising the need for sub-category analysis when assessing these products.

Although the benefits of substituting meat with PBMAs from an environmental perspective are well documented in the literature [32,42,43,44,45,46], the packaging used for PBMAs is often overlooked. Sustainable packaging has been reported to be important to consumers of plant-based products in the UK and Republic of Ireland (ROI) [47]. Therefore, this study aims to use information on food labelling to monitor and report changes in packaging recyclability over time of PBMAs available on the island of Ireland (IOI) to determine whether consumer demand for more sustainable packaging materials for PBMAs is being met by the food industry.

In an online consumer study of 456 plant-based consumers and non-consumers in the UK and ROI, ‘Product information on the label’ and ‘Ingredient list’ jointly scored the second highest for attributes of importance after ‘Taste’ for plant-based consumers when selecting these products [47]. Considering the importance of these attributes to consumers in this context, this study aims to collect and evaluate all information on the packaging of PBMAs available on the Island of Ireland (IOI) at three different timepoints. Whilst several studies have utilised on-pack information for PBMAs available in the UK or IOI already [10,18,29,32,33,34,36,39,43], these studies have mostly extracted information on nutrient content only and at one timepoint. Given the importance to consumers of other non-nutrient product information on-pack such as ingredients lists and packaging materials, this study aims to provide a more holistic view of PBMAs. Considering the pace of new product development within this rapidly growing market, analyses for single timepoints may become quickly outdated. Therefore, this study aims to address this limitation of previously published studies by capturing data at three timepoints to determine changes in PBMAs available on the IOI from 2021 to 2023 with regard to product offering, nutritional composition, ingredients, claims and packaging recyclability. In addition to this, this study aims to compare the nutritional quality of PBMAs available in 2023 with similar categories of meat equivalent products.

## 2. Materials and Methods

### 2.1. Data Collection

Three product audits of PBMAs available online in Sainsbury’s and Tesco supermarkets were conducted in February–April 2021 and November–January 2022/2023 and 2023/2024. The two supermarket retailers were selected due to their online presence on the IOI and joint market share, accounting for over 40% of the UK market [48]. Category filters such as ‘meat free’ and ‘vegetarian’ were used to identify PBMAs on the retailer websites. Products created to mimic or replace conventional meat products were included, whilst naturally meat-free (MF) products such as margarita pizza and vegetable curry were excluded in the audit.

### 2.2. Data Extraction

On-pack information was extracted from the supermarket websites and inputted into four inter-related Microsoft^®^ Excel^®^ databases (Microsoft 365, Version 2408). This included the product name, brand and description, the unit weight, portion size, country of origin, nutritional content per 100 g (cooked or as sold depending on what was stated on the webpage), ingredients and sub-ingredients, allergens, claims, packaging material and recyclability. If a product was sold by both retailers, it was logged only once on the database. Products were grouped according to their similarity and likeness to conventional meat products.

Following each audit, a quality control check of the databases was performed, whereby extracted data for 10% of products (selected at random) at each timepoint were cross-checked with the information on the retailer websites.

### 2.3. Nutritional Assessment

The nutritional composition of PBMAs across the different timepoints was assessed through the calculation of median energy and nutrient contents per 100 g for the different product categories. A-scores from the UK’s Nutrient Profiling Model [49] were allocated to all products and median A-scores for each product category were compared for each timepoint. The A-score is a composite score derived from a sum of points allocated for the energy (kJ), saturated fat (g), total sugar (g) and sodium (mg) content per 100 g of food. A maximum of 10 points per nutrient can be allocated, with the number of points awarded dependent on the thresholds set out in the nutrient profiling technical guidance document [49]. Thresholds from the UK’s front of pack (FoP) traffic light labelling system were applied to classify products as ‘low’, ‘medium’ or ‘high’ in total fat (≤3 g/100 g, >3 to ≤17.5 g/100 g and >17.5 g/100 g), saturated fat (≤1.5 g/100 g, >1.5 g to ≤5 g/100 g and >5 g/100 g), sugars (≤5 g/100 g, >5 g to ≤22.5 g/100 g and >22.5 g/100 g) and salt (≤0.3 g/100 g, >0.3 to 1.5 g/100 g and >1.5 g/100 g) [50]. Since the nutrients included in the UK’s A-score and FoP traffic light labelling system are nutrients to limit, lower numbers are considered better from a nutritional perspective for these specific nutrients and composite score. EU thresholds for nutrient claims were applied to determine the proportion of products within each year and category which could be considered ‘a source of’ or ‘high in’ protein (≥12% or ≥20% energy from protein), iron (≥2.1 mg and ≥4.2 mg/100 g) and vitamin B12 (≥0.38 μg and ≥0.75 μg per 100 g) [51]. For micronutrient analysis, micronutrients for which ≥3 products reported contents at two or more timepoints were included. Products were also grouped according to their main protein source and percentage of energy from protein (<12%, ≥12% and ≥20% energy from protein).

### 2.4. Ingredient Analysis

To determine the most frequently occurring ingredients, a frequency analysis of the main ingredients (excluding sub-ingredients), allergens and additives present in products at each timepoint was conducted. Allergens were identified according to the 14 major allergens recognised by law in the EU [52]. The main protein ingredient(s) of included products (except for the pizza category), was/were identified through the product description. In the absence of the protein source in the product description, the ingredients listings were checked to identify the main protein source. Products were then checked manually using search terms in the product description to ensure products were grouped according to the most appropriate protein source. For meals such as ‘Quorn spiced chickpea & lentil bowl’ which contained Quorn pieces plus legumes and vegetables, the main protein source of the PBMA, i.e., the Quorn pieces, was used. The degree of processing of the products was determined by applying the NOVA classification based on ingredients listed [53]. This classification system is the most commonly applied in the scientific literature [53].

### 2.5. Comparison with Meat Products

Products identified in the 2023 audit were filtered to include PBMA categories containing products specifically designed to imitate meat. Categories included in the analysis for this section are ‘Burgers’, ‘Sausages’, ‘Chicken’, ‘Beef’, ‘Seafood’, ‘Bacon/Slices’, ‘Pastry/Pies’ and ‘Mince’. McCance and Widdowson’s 2021 Composition of foods integrated dataset (CoFID) [54] was used to source nutritional data per 100 g (cooked and uncooked) of meat equivalent products within the same product categories outlined above. Filters were applied to the CoFID dataset to identify products which corresponded to the identified categories.

### 2.6. Statistical Analysis

Number and percentages are reported for data related to changes in PBMAs over time. Descriptive data are reported as median and range or mean and standard deviation (SD) depending on data distribution. Continuous data were checked for normality using Shapiro–Wilk tests. For normally distributed data (*p* > 0.05), independent samples *t*-tests were used, and for non-normally distributed data, Mann–Whitney U tests were used. Depending on data distribution, the parametric and non-parametric tests were used to compare PBMAs with comparable meat products, with a *p* value < 0.05 considered statistically significant. SPSS^®^ statistical software (IBM SPSS Statistics, Version 26.0, Armond, NY, USA) and Microsoft^®^ Excel^®^ (Microsoft365, Version 2408) were used for statistical analysis.

## 3. Results

### 3.1. Assessment of the Market

**Figure 1:**
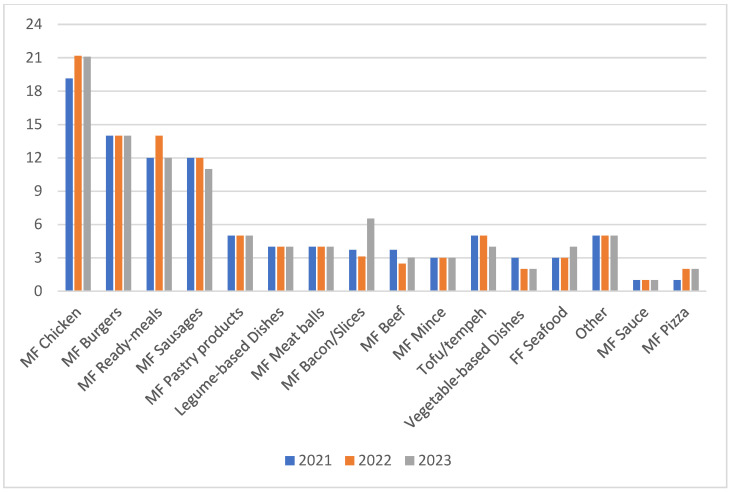
The percentage of products within each category as a proportion of the total products on sale at each timepoint. MF: meat-free.

A total of 350, 321 and 398 products were included in the 2021, 2022 and 2023 audits, respectively, with a 9% decrease in the total number of PBMAs available in 2022 vs. 2021 and a 14% and 19% increase in the number of products available in 2023 vs. 2021 and 2023 vs. 2022, respectively. Products were grouped together into 1 of 16 categories based on their likeness to meat equivalent products. A description of the categories is provided in Supplementary Table S1. The ‘MF Chicken’, ‘MF Burgers’, ‘MF ready-meals’ and ‘MF Sausages’ categories contained the most products, accounting for approximately 60% of all products at each timepoint (Figure 1). The percentage of products within each category at each timepoint was similar, with the exception of the ‘MF Chicken’ and ‘MF Bacon/slices category’, in which there was around a 25% and 100% increase in the number of products in these categories in 2023, respectively, compared to 2021 numbers (Figure 1).

The number of brands in the market increased over time with 47, 52 and 61 different brands observed in 2021, 2022 and 2023, respectively (Supplementary Table S2). Despite this growth, Quorn™, Tesco Plant Chef, Sainsbury’s Plant Pioneers and Linda McCartney dominated the market at all three timepoints, collectively accounting for 43%, 41% and 45% of all products in 2021, 2022 and 2023, respectively.

### 3.2. Changes in the Nutritional Composition of PBMAs over Time

Median energy and nutrient contents of the different product categories mostly remained similar over time (Supplementary Table S3). All products in the ‘MF Beef’, ‘MF Meatball’, ‘MF Mince’ and ‘MF Sauces’ categories contained ≥20% energy from protein at all three timepoints (Table 1). For the ‘MF Burgers’, ‘MF Bacon/slices’, ‘MF Tofu/Tempeh’ and ‘Other’ categories, the proportion of products containing ≥20% energy from protein increased over time by 7%, 12%, 17% and 3%, respectively, for 2023 vs. 2021 products. For the ‘MF Pastry’, ‘MF Seafood’ and ‘MF Pizza’ categories, the number of products with <12% energy from protein increased over time with almost 50%, 70% and 90% of the products in these categories in 2023 containing <12% energy from protein. None of the products in the ‘Vegetable-based dishes’, ‘Legume-based dishes’ and ‘MF Pizza’ categories contained products with ≥20% energy from protein at any timepoint.

Median A-scores in 10/16 categories were lower in the 2023 vs. 2021 audit, with reductions of 0.3–1.4 points observed (Table 1). Median A-scores for the ‘MF Bacon/Slices’, ‘MF Ready-meals’, ‘Other’, ‘Vegetable-based dishes’, ‘Legume-based dishes’ and ‘MF Sauce’ categories increased by 0.1–1.7 points in 2023 compared to 2021 products.

The most declared micronutrients on the nutrition information panel (NIP) were vitamin B12 and iron at all three timepoints. An increase in the number of products declaring these micronutrients on-pack over time was observed, with around 7–8% of products declaring vitamin B12 and iron contents on-pack in 2021 and 2022 and 10% of products in 2023 declaring vitamin B12 and iron contents on-pack. Of the 5 eligible categories included in the micronutrient analysis (‘MF Burgers’, ‘MF Bacon/slices’, ‘MF Chicken’, ‘MF Beef’ and ‘Other’), the median content of vitamin B12 and the number of products within these categories declaring vitamin B12 content on-pack increased over time in 3/5 categories (Table 2). The median iron content increased over time across all four categories (100%) included in the iron analysis (‘MF Burgers’, ‘MF Bacon/Slices’, ‘MF Chicken’ and ‘MF Beef’). The number of products eligible to make ‘high in’ claims for iron and vitamin B12 was the highest in the 2023 audit for all categories except for the ‘MF Bacon/slices’ category for vitamin B12.

### 3.3. Changes in Ingredients, Allergens and Additives

For the three timepoints, ‘Soy’, ‘Mycoprotein’, ‘Pea’ and ‘Soy and wheat’ combined were the most used protein sources, present collectively in 63% of the products at each timepoint with a slight reduction in the number of mycoprotein-based products in 2022 (n = 36, 11%) compared to 2021 (n = 54, 16%) and 2023 (n = 71, 18%). When products were compared according to their percentage of energy from protein, products with protein sources from ‘Soy,’ ‘Mycoprotein,’ ‘Pea,’ and ‘Soy and wheat’ were more likely to contain ≥12% or ≥20% energy from protein for all three timepoints (Supplementary Table S4). Of the products with <12% energy from protein at each timepoint, around 20% were vegetable-based and 13% were made from jackfruit. Mushroom-based products made up 14%, 7% and 5% of products with <12% energy from protein in 2021, 2022 and 2023, and pea-based product made up 13% of products with <12% energy from protein in 2021 and 2022 and 8% in 2023 (Supplementary Table S4).

The median number of main ingredients (excluding sub-ingredients) increased from 2021 to 2023 for the ‘MF Pastry’ (15.3 vs. 19.4) ‘MF Bacon/Slices’ (21.1 vs. 16.4), ‘MF Mince’ (11.7 vs. 14.8), ‘MF Chicken’ (14.8 vs. 18.0) and ‘MF Sauce’ (9.4 vs. 13.5) categories, with an increase of 3–4 ingredients on average within these categories from 2021 to 2023 (Table 3). The median number of main ingredients in the ‘Vegetable-based dishes’ (24.7 vs. 21.0) and ‘Legume-based dishes’ (29.3 vs. 22.1) decreased by 4 and 7, respectively, in 2021 vs. 2023. The category with the lowest average number of main ingredients was the ‘Tofu/Tempeh’ category with a median of around six ingredients at each timepoint.

The percentage of products with allergens reduced substantially between 2021 and 2022 (−34%); however, between 2022 and 2023, this had increased from 58% of products to 79% of products in 2023, which was still a reduction from the 92% of products with allergens in 2021. Wheat (cereals containing gluten) was the most common allergen found in PBMAs with 58–60% of allergens declared on-pack across the different timepoints being wheat. Soya was the next most declared allergen on pack, accounting for 22–26% of allergens declared across the different timepoints. Eggs and milk were the next most frequently declared allergens on-pack, accounting for 4–5% and 2–6% of allergens declared across the timepoints.

The proportion of products in the ‘MF Sausages’, ‘MF Mince’ and ‘FF Seafood’ categories with ≥1 allergen, decreased over time by 5%, 9% and 29%, respectively, from 2021 to 2023, whilst the number of products containing allergens in the ‘MF Bacon/Slices’, ‘MF Chicken’, ‘MF Meatballs’, ‘Legume-based dishes’ and ‘MF Pizza’ categories increased over time by 19%, 9%, 14%, 17% and 25%, respectively, from 2021 to 2023 products (Table 3). At the three timepoints, the ‘Vegetable-based dishes’ category contained the fewest number of products with allergens (22%, 29% and 14% of products with allergens in 2021, 2022 and 2023, respectively). Apart from the ‘MF Pizza’ category, the category with the highest proportion of products with allergens at each timepoint was the ‘MF Pastry’ category, of which 79%, 80% and 74% of products within this category in 2021, 2022 and 2023, respectively, contained ≥1 allergen.

A total of 900, 903 and 995 different ingredients were included in the ingredients lists of products in the 2021, 2022 and 2023 audits, respectively. Approximately 80% of the ingredients included in PBMAs at each timepoint were mentioned ≤5 times, with around 47% of ingredients mentioned only once at each timepoint. Salt, water and rapeseed oil were the top 3 most frequently listed ingredients on products at the three timepoints, with the top 10 most frequently used ingredients the same across the three timepoints (Table 4). Methylcellulose and calcium carbonate were the top 2 most frequently used additives at all three timepoints, with a range of other additives used across the three timepoints. Products were assigned NOVA scores of ≥3 at each timepoint, with 91%, 92% and 90% of products in 2021, 2022 and 2023 assigned a NOVA score of 4, thereby classifying these products as UPFs [53].

### 3.4. Claims

Nutrition claims were present on 54% of products in 2021 and 2022 and 61% of products in 2023, with 49%, 44% and 37% of these products in 2021, 2022 and 2023, respectively, containing two nutrition claims. Of the total number of products containing claims in 2021, 2022 and 2023, 97%, 95% and 90%, respectively, had a NOVA score of 4, classifying these products as ultra-processed foods. For the three timepoints, claims concerning protein, fibre and saturated fat content were the most used nutrition claims, present on 87–89%, 37–46% and 28–33% of products for protein, fibre and saturated fat, respectively. There was a slight reduction in the number of fibre-related claims (−9%) and slight increase in the number of saturated fat claims (+5%) in 2023 vs. 2021 products. Micronutrient-related claims were mostly regarding iron and vitamin B12, with ≤4 products at each timepoint containing claims related to vitamin A, calcium, folic acid, magnesium, phosphorus and zinc, whilst 16%, 14% and 12% of products making claims in 2021, 2022 and 2023, respectively, were related to iron and 11–12% of products making claims in 2021, 2022 and 2023 were related to vitamin B12 (Supplementary Table S5).

Of the products with claims related to protein, 18–21% were considered high in total fat, saturated fat, sugars or salt across the timepoints, according to the UK FoP labelling thresholds [50]. Between 10 and 17% of the products making fibre-related claims contained high contents of at least one of these nutrients and 6–15% of products making a low in saturated fat claim, were high in total fat, sugars or salt (Supplementary Table S5).

Almost all products at each timepoint contained at least one lifestyle claim, and this claim rate has increased over the years, rising to 96% of products in 2023 compared to 91% in 2021. Around a third of products at each timepoint with lifestyle claims contained two claims. Claims around suitability of the products for vegans were the most used and were present on 58%, 68% and 75% of products in 2021, 2022 and 2023, respectively (Supplementary Table S6). Claims around the suitability of the product for vegetarians were the next most used, present on 47%, 39% and 37% of products with health/lifestyle claims in 2021, 2022 and 2023, respectively. Gluten-free claims were present on 17%, 14% and 12% of products in 2021, 2022 and 2023, respectively, whilst the number of products containing ‘plant-based’ claims has increased over time from 14% of products with claims in 2021 to 20% of products with claims in 2023.

Of the 37 different lifestyle claims made, 7 (19%) were related to natural ingredients or a clean label. These included claims such as no artificial colours/flavours/preservatives/additives, ‘all natural ingredients’, ‘organic’ and ‘no junk’, all of which were made on a small proportion of the products (≤6%) at each timepoint.

### 3.5. Recyclability of Packaging

At each timepoint, around 60–70% of products were made from packaging materials that were partially recyclable. Only 21%, 29% and 23% of products in 2021, 2022 and 2023, respectively, could be fully recycled at home. The proportion of products at each timepoint which could be recycled at large supermarkets was between 7 and 8%. Less than 3% of products at each timepoint were made from materials not suitable for recycling, as well as ≤3% of products stated online for consumers to check the local recycling rules before recycling. Between 20 and 27% at each timepoint did not specify online the recyclability of the product packaging.

### 3.6. Nutritional Comparison with Meat

Included in the comparative analysis with meat products were 270 PBMAs and 82 meat equivalent products grouped into eight categories: ‘Burgers’, ‘Sausages’, ‘Bacon/Slices’, ‘Mince’, ‘Seafood’, ‘Beef’, ‘Pastry/Pies’ and ‘Chicken’. PBMA ‘Burgers’, ‘Sausages’ and ‘Bacon/Slices’ contained significantly lower calories than comparable meat products (Table 5). Average protein and total fat contents of PBMAs were significantly lower than comparable meat products in 4/8 and 5/8 categories, respectively. Only PBMAs in the ’Chicken’ category contained significantly higher total fat contents than comparable meat products (9.5 (5.8–14.5) vs. 3.9 (2.4–6.0), *p* = 0.01). Similarly, the saturated fat content of PBMAs was significantly lower than meat for 6/8 categories. The carbohydrate, sugar and salt contents of PBMAs were significantly higher than comparable meat products for 6/8, 5/8 and 4/8 categories, respectively. Only PBMAs in the ‘Bacon/Slices’ category contained a significantly lower salt content than meat-equivalent products (2.2 ± 0.8 vs. 3.5 ± 0.7, *p* < 0.001). PBMAs contained significantly higher fibre contents than meat products in 6/7 categories included in the fibre analysis (‘Burgers’, ‘Sausages’, ‘Bacon/Slices’, ‘Mince’, ‘Seafood’ and ‘Chicken’).

PBMAs had a significantly lower average A-score than meat products in the ‘Burgers’ (7.5 (5.0–9.0) vs. 12.0 (10.3–17.5), *p* < 0.001), ‘Sausages’ (8.0 (7.0–10.0) vs. 17.0 (11.0–17.0), *p* < 0.001), ‘Bacon/Slices’ (11.0 ± 3.8 vs. 20.1 ± 3.5, *p* < 0.001), ‘Beef’ (6.9 ± 2.0 vs. 14.8 ± 0.9, *p* < 0.001) and ‘Pastry/Pies’ categories (12.7 ± 2.7 vs. 20.6 ± 2.2, *p* < 0.001) (Table 5).

PBMAs contained fewer products ‘high’ in total and saturated fat than meat products across all categories, except for the ‘Chicken’ category, where a small proportion of PBMAs were ‘high’ in total fat compared to 0% of the meat products. However, the reverse was observed for saturated fat within this category (Supplementary Figure S1). Most PBMAs and meat products were low in sugar, with a small percentage of products in the ‘Seafood’ (13% and 7% of PBMA and meat products, respectively), ‘Chicken’ (5% of PBMAs) and ‘Bacon/slices’ (5% of meat products) categories having medium or high sugar contents. For most categories, a higher proportion of PBMAs contained medium or high salt content compared to meat products, with the exception of the ‘Sausages’ and ‘Bacon/slices’ categories in which 45% vs. 20% and 100% vs. 77% of meat products and PBMAs in the ‘Sausages’ and ‘Bacon/slices’ categories, respectively, were high in salt.

PBMAs in the ‘Burger’, ‘Chicken’ and ‘Seafood’ categories contained fewer products, with ≥20% energy from protein compared to meat products within the same categories (Supplementary Figure S2). PBMAs in the ‘Sausage’ and ‘Pastry’ categories contained more products with ≥20% and ≥12% energy from protein, respectively, and less products with less than <12% energy from protein compared to the meat products within these categories. All PBMAs and meat products in the ‘Mince’ and ‘Beef’ categories and most products in the ‘Bacon/slices’ category contained ≥20% energy from protein.

## 4. Discussion

The number of PBMAs available in 2023 has increased compared to 2021 numbers, with the ‘Chicken’, ‘Burger’, ‘Meals’ and ‘Sausages’ categories the largest at all timepoints. While the number of brands of PBMAs increased over time, the same four brands dominated the market at each timepoint (Quorn, Sainsbury’s, Tesco’s and Linda McCartney). Slight improvements in the nutrient profiles of PBMAs over time were detected with regard to reduced A-scores and increased fortification with iron and vitamin B12. The main protein sources used for PBMAs were ‘Soy’, ‘Mycoprotein’, ‘Pea’ and a combination of ‘Soy and wheat’, accounting for around 60% of products at each timepoint. Whilst a slight reduction in the percentage of products containing ≥1 identified allergen was observed in 2022 (58%) and 2023 (79%) vs. 2021 (92%), most PBMAs available on the IOI contain ≥ 1 allergen. Around 90% of PBMAs at each timepoint were classified as UPFs according to NOVA taxonomy [53], with methyl cellulose and calcium carbonate the most frequently listed additives on-pack across the three timepoints. The presence of nutrition claims on PBMAs has increased over time (54% of products in 2021 and 2022 and 61% in 2023), with most nutrition claims relating to protein at all timepoints (87–89%). Almost all PBMAs at each timepoint contained a lifestyle-related claim (91–96%) with an increase in the use of claims related to suitability for vegans (58%, 68% and 75% in 2021, 2022 and 2023, respectively). While PBMAs are often marketed as being better for the planet or having a low environmental impact, only 21–29% of products across the three timepoints were made from packaging which could be fully recycled at home.

For most categories, PBMAs had significantly lower total and saturated fat content and higher carbohydrate, fibre and sugar contents than meat products within the same category. Alternatives to red and processed red meat categories such as ‘Burgers’, ‘Sausages’, ‘Bacon/Slices’, ‘Beef’ and ‘Pastry/pies’ had significantly lower mean A-scores and therefore contained less nutrients to limit.

### 4.1. PBMAs: Changes over Time

The increase in the number of PBMAs available on the IOI in recent years perhaps reflects the increasing number of self-declared vegans and vegetarians in the UK over a similar time period [55], as well as significant increase in the reported consumption of PBMAs over the last 10 years captured in UK and Irish food consumption data [14,15]. The largest product categories (‘MF Chicken’, ‘MF Burger’, ‘MF Meals’ and ‘MF Sausage’) identified are consistent with a 2020 online audit conducted by *safe*food on PBMAs available on the IOI across four supermarkets (Tesco, Sainsbury’s, Asda and SuperValu) [10]. The higher proportion of products within the above-mentioned categories could be explained by the increased feasibility to replicate the taste, texture and appearance of processed meat products than prime meat cuts such as steak and lamb [11].

There were no noteworthy changes in the energy or macronutrient content of PBMAs over time, except for a decrease in median A-scores for most categories, indicating slight improvements in the nutritional profiles of these products. With personal health as one of the key determinants for consumers entering into the PBMA market [7,10,47,56] and a ‘health halo’ surrounding PBMAs [57], reformulations and innovation to improve the nutritional profile of these products are important. The widespread use of nutrition and lifestyle claims on PBMAs are likely contributing to the ‘health halo’ surrounding these products [10,13,22,34,58]. Considering that 90–97% of PBMAs at each timepoint carrying a nutrition claim were considered UPFs based on the NOVA classification [53] and over a third of PBMAs with protein-related nutrition claims contained high amounts of either total fat, saturated fat, sugar or salt, based on thresholds from the UK government’s FoP traffic light labelling system [50], it is evident that the use of such claims to promote nutrients such as protein, may mask the high levels of nutrients to limit within some products.

Protein content has been cited as a barrier to consumption of PBMAs for some consumers [56,59]. Whilst protein is not considered a nutrient of concern for generally healthy adults in high income countries where PBMAs are often consumed [60], consumers may be justified in their concerns around the protein content of PBMAs, with 16–18% of PBMAs at each timepoint not considered a source of protein based on EU Commission thresholds for protein-related claims [51]. Although this figure is much lower than the 28% reported in a previous audit of PBMAs on the IOI [10], it highlights the importance of consumer knowledge when selecting PBMAs, particularly since these products are likely to be used as the protein component of a meal [61].

Protein content and source have been identified as important factors in the selection of plant-based alternatives by consumers [7,56,59,62], with transparency of the protein source on ingredients listings found to be more positively received among Danish consumers [62]. ‘Soy’, ‘Mycoprotein’, ‘Pea’ or a combination of ‘Soy and wheat’ were identified as the most used protein sources in the present audits and in previous studies [17,20,21,22,24,26,30,31,34,63]. Whilst the use of soy as a protein source is common in PBMAs, this ingredient was perceived negatively in terms of health and the environment among participants in New Zealand [56]. Whilst negative perceptions of soy from an environmental perspective may be justified due to soy production being a key driver of deforestation in Brazil and consequent habitat and biodiversity loss [64,65], steps have been taken through the UK Soy Manifesto to ensure that soy imported to the UK is deforestation- and conversion-free [66]. Reducing the environmental impact of soy through responsible sourcing and transparent supply chains is an important step since soy is a nutritionally dense food, with most soy-based PBMAs identified in the current study as containing protein contents sufficient to be considered sources of, or high in, protein according to EU legislation on protein-related nutrient claims [51]. Other studies have reported soy-based PBMAs to contain higher amounts of fibre, omega-3 fatty acids and micronutrients such as iron, zinc, B vitamins and folic acid compared to PBMAs from other protein sources [67,68]. Therefore, soy appears to be an acceptable protein source for PBMAs; however, reducing the environmental impact of this ingredient is essential to future-proof its use in these products. The large number of products with mycoprotein as a protein source could be context-specific considering that Quorn, the manufacturer of mycoprotein products, is a UK-based company and is well established in UK and Irish markets [7]. Familiarity with the Quorn brand perhaps provides an explanation for why more UK and Irish consumers reported being open to consider mycoprotein foods than French, Dutch, German and American consumers based on an online consumer survey of 6077 participants [7].

The number of PBMAs fortified with vitamin B12 and iron has increased over time; however, this is still limited with only 10% of products in 2023 declaring the content of these micronutrients on-pack. The level of fortification has also increased, with more products eligible to make ‘high in’ claims for iron and vitamin B12 in 2023 compared to previous years [51]. Since the fortification of PBMAs is not mandatory, the scale of fortification of these products differs across countries and markets [13,20,30,35]. Since meat is an important contributor to intakes of a range of micronutrients such as vitamin B12, iron, zinc and selenium [69,70,71] and micronutrient deficiencies continue to persist in the UK and Ireland, with around 11% of women aged 15–49 years old living with anaemia and no progress made to reduce prevalence rates of anaemia [72], the use of fortification should be more widespread within the PBMA sector and the use of processing techniques such as soaking beans and grains to reduce phytic acid and increase iron and zinc bioavailability [73,74]. Therefore, the ability of PBMAs to address rather than exacerbate micronutrient deficiencies through fortification presents a major opportunity for the PBMA sector and public health.

This study uses data available on-pack only; therefore, it is possible that more PBMAs contain micronutrients that are not declared on NIPs. This is known for mycoprotein-based products, which, based on European Commission nutrient claims thresholds, are ‘high in’ zinc, selenium, phosphorous, manganese, copper and chromium [75]. However, none of the Quorn products identified in the audits listed micronutrient content on-pack since manufacturers are not required by law to declare this when the product has not been fortified [76]. Whilst analysing NIPs provides useful insights into the nutritional profiles of these products, further research using laboratory analytical methods, in vitro studies and human trials would be useful to better understand the micronutrient contents of PBMAs, the impact of the food matrix on nutrient absorption and the contribution of PBMAs to nutrient intakes and status.

Salt was the most frequently used ingredient in PBMAs identified in the three audits. This is consistent with findings from our previously published systematic review of PBMAs, in which 88% of the products identified contained added salt [63]. In addition to its use as a flavour enhancer, salt is added to PBMAs for functional purposes, including textural and preservative purposes [77]. To formulate products with meat-like textures, hydrocolloids are often used, particularly in the absence of wheat gluten [77]. Hydrocolloids are a group of polymers that have gelling, thickening and stabilising functions used to create a fibrous, meat-like texture [77]. The top 10 frequently used additives found in the ingredients lists of the PBMAs identified across the three audits included hydrocolloids, namely methyl cellulose, carrageenan, pectins, xanthan gum and sodium alginate. For the optimum functionality of hydrocolloids, appropriate concentrations of salt are required [77]. Therefore, although reformulations to reduce the salt content of PBMAs is often recommended to improve the nutritional profile of these products, this may negatively affect the sensory properties of PBMAs. With taste and texture key attributes in consumer acceptability of PBMAs [57,78], further work is needed to balance improvements in nutritional profile and advancements in the sensory properties of these products. Research and innovation to refine and improve PBMAs from a nutritional, sensory, affordability and sustainability perspective is planned for the National Alternative Protein Innovation Centre (NAPIC) in the UK [79] and is underway at Stanford University [80]. Therefore, future PBMAs will hopefully be improved as a result of the ongoing research and innovation in this sector.

Despite consumer demands for cleaner labels on PBMAs well established in the literature [7,62], additives such as methyl cellulose and calcium carbonate appeared frequently in the PBMAs identified in this study and in previous studies [17,26]. The frequent use of calcium carbonate in PBMAs could explain the higher calcium contents of PBMAs compared to meat [63] and increased calcium intakes in modelling studies where meat is replaced with PBMAs [81,82]. Although additives play important functional roles in foods as mentioned above, reformulation to make PBMA labels ‘cleaner’ and remove ingredients identified as common allergens could help producers/manufacturers meet consumer demands and likely increase sales [7,62]. However, the feasibility of this without compromising on taste is likely to prove challenging.

Most products identified in this audit were classified as UPFs according to the NOVA classification system [53]. This finding is consistent with the findings of other cross-sectional and audit studies, whereby the majority of PBMAs were assigned a NOVA score of 3 or 4, i.e., processed or ultra-processed, respectively [22,30,32,42,82,83]. Whilst a range of negative health outcomes have been associated with excess consumption of UPFs, randomised controlled trials (RCTs) investigating the impact of PBMA consumption have not found any associations with adverse health effects [84,85,86,87,88,89,90,91,92]. However, these studies are short-term; therefore, longer-term studies are needed to ensure the introduction of these products into habitual diets will not result in any adverse health outcomes.

The use of sustainable packaging for plant-based products scored a 4 on a 5-point Likert scale by 456 participants in the UK and ROI, indicating that participants considered this important [47]. Recyclable packaging was voted the most resonant on-pack messaging by 38% of participants in a large online consumer survey of 6077 participants across four European countries, the UK and the U.S.A [7]. Therefore, the use of recyclable materials for the packaging of PBMAs is important for consumers, yet the findings from the current study revealed that less than a third of products at each timepoint were eligible to be fully recycled at home. The widespread use of a clear film on PBMA packaging to allow visibility of the product means packaging containing this material is only partially recyclable. With product visibility important to consumers of PBMAs, this presents a challenge for manufacturers to tackle these conflicting consumer demands.

### 4.2. Nutritional Assessment of PBMAs and Meat

For most categories, PBMAs had significantly lower total and saturated fat content and higher carbohydrate, fibre and sugar content than meat products within the same category. These differences in nutritional contents are consistent with findings from our previously conducted systematic review [63], as well as a systematic review by Nájera Espinosa et al. (2024) [93]. Slight variation between different product categories was observed in this study, with alternatives to red and processed red meat products, such as ‘Burgers’, ‘Sausages’, ‘Bacon/Slices’, ‘Mince’ and ‘Pastry/pies’, containing significantly lower total and saturated fat content. However, alternatives to ‘Chicken’ were significantly higher in total and saturated fat content compared to meat equivalents, whilst non-significant differences were observed for the seafood category. Similar findings were reported in a nutritional assessment of PBMAs and comparable processed meat products available in UK supermarkets in 2022, whereby PBMAs were considered ‘healthier’ when compared with processed red meat, but not when compared with poultry or seafood [34]. ‘Healthiness’ was assessed in this particular study using the UK’s Nutrient Profiling Model (NPM) [49]. Whilst considerable variability in the nutritional profile of PBMAs exists, identifying healthier swaps could be used to better inform consumers when making dietary changes to reduce their meat intakes.

Total fat, saturated fat and fibre are nutrients of concern within the UK population, with 54% and 78% of the population estimated to exceed the recommended limits for daily total and saturated fat, respectively [94]. The mean daily intake of dietary fibre for UK adults on average is approximately 11 g lower than the recommended intake of 30 g/day [95]. Partial displacement of meat in diets, particularly red and processed meat with PBMAs, could help address excessive intakes of total and saturated fat and inadequate intakes of fibre. Diets that are high in saturated fat and low in dietary fibre are associated with a higher risk of cardiovascular diseases, which are a leading cause of disability adjusted life years (DALYs) and mortality in the UK and Ireland [96,97]. Therefore, dietary changes that positively impact intakes of these macronutrients could result in public health benefits.

The higher salt/sodium content of PBMAs compared to meat in 4/8 categories is an important nutritional shortcoming of these products, with the overconsumption of sodium a key dietary contributor to the global burden of disease [98]. Considering the already-high salt/sodium intakes in countries where PBMAs are more commonly consumed [98], replacing meat in the diet with PBMAs could increase salt/sodium intakes as predicted in modelling studies exploring the impact of meat substitution with PBMAs on nutritional intakes or prevalence of nutritional inadequacies [81,82,99]. However, these findings do not account for salt use during cooking or the addition of table salt to PBMAs vs. meat products, in which practices around salt use might differ for PBMA and meat consumption. A crossover RCT in which 36 participants in the U.S.A consumed ≥2 servings/day of PBMAs vs. ≥2 servings/day of animal meat for 8 weeks whilst keeping all other aspects of the diet the same did not detect a significant difference in sodium intakes between the two diet phases [84]. This could be explained by the addition of sodium to raw meat accounted for in the dietary intake assessments [84]. Although a better understanding of salt use when preparing and eating meat and PBMAs would be valuable in determining whether differential salt contents of PBMAs and meat result in increased salt intakes, reformulation to reduce the salt content of PBMAs should be a key priority for the food industry.

The present study is the first to holistically assess PBMAs available on the IOI at three timepoints during a period of rapid market growth and to compare the nutritional content and quality of PBMAs with meat equivalent products. A range of product categories were outlined in the analysis, which provides a better understanding of the PBMA categories and tend to have better nutritional profiles, as well as the meat substitutions which may have positive impacts on nutrient intakes. This study highlights the strengths and shortcomings of these products in terms of nutrient contents, ingredients and packaging, which may be useful for food manufacturers. However, this study also has some limitations, including the exclusion of major retailers and smaller-scale supermarkets on the IOI in product audits. The inclusion of additional leading retailers could allow for a more accurate representation of the PBMA market for this context. This study calculated the A-score only, as opposed to the full NPM score due to data limitations. However, calculating the full NPM scores would enable the classification of PBMAs and comparable meat products as ‘healthy’ and ‘less healthy’ based on thresholds outlined in the UK government’s NPM technical guidance document [49]. This study did not conduct a price comparison of PBMAs and meat equivalent products. Considering that affordability is an important dimension of a defined healthy and sustainable diet [100], understanding price differences would be useful for assessing the potential role of PBMAs in healthy and sustainable diets as well as the accessibility of these products for lower-income households on the IOI.

## 5. Conclusions

An increase in the number of products available in 2023 compared to previous years suggests that the development of new products in the PBMA market is still ongoing. With only minor improvements to the nutritional profile of PBMAs observed over time, further fortification and reformulation, particularly to increase iron, zinc and vitamin B12 contents while reducing salt contents, would enhance these products. Although the proportion of products containing ≥1 common allergen has reduced over time, the presence of allergens within these products is still widespread, making the majority of PBMAs inaccessible for a subset of the population with allergies. The enhanced use of fully recyclable packaging materials is needed to satisfy consumer demands and reduce the environmental impact of these products. Substituting processed meats such as ‘Bacon/Slices’, ‘Sausages’ and ‘Pastry/Pies’ with PBMAs has the potential to impact macronutrient intakes more positively than substituting less processed meats such as ‘Beef’, ‘Chicken’ and ‘Fish’. Improved guidance for consumers on which meats to substitute with PBMAs, along with criteria for selecting PBMAs of superior nutritional quality, would be valuable for mitigating potentially negative nutritional trade-offs when replacing meat with PBMAs in the diet. This study contributes to the existing literature in this field by providing a holistic view of PBMAs available on the IOI. Further research into fortification strategies and appropriate salt substitutes is needed to enhance the nutritional profile of these products without compromising taste, texture or quality. In addition, research into the development of innovative and recyclable packaging materials for these products is needed.

## Figures and Tables

**Table 1:** Average A-scores (median and range) and number and percentage of products within each category at each timepoint with <12%, ≥12% and ≥20% energy from protein.

	Year, n	A-Score *	<12% Energy from Proteinβ	≥12% Energy from Proteinβ	≥20% Energy from Proteinβ
MF Burgers	2021 (n = 49)	8.4 (4–19)	18 30.6%	7 14.3%	27 55.1%
	2022 (n = 44)	8.0 (4–19)	15 34.1%	6 13.6%	23 52.3%
	2023 (n = 56)	7.8 (5–18)	14 25%	7 12.5%	35 62.5%
MF Sausages	2021 (n = 42)	9.1 (5–17)	1 2.4%	7 16.7%	34 81%
	2022 (n = 40)	8.6 (5–17)	1 2.5%	9 22.5%	30 75.0%
	2023 (n = 45)	8.8 (5–18)	2 4.4%	5 11.1%	38 84.4%
MF Pastry products	2021 (n = 19)	13.6 (9–17)	7 36.8%	11 57.9%	1 5.3%
	2022 (n = 15)	12.7 (5–17)	5 33.3%	9 60.0%	1 6.7%
	2023 (n = 19)	12.7 (5–18)	9 47.4%	9 47.4%	1 5.3%
MF Bacon/slices	2021 (n = 13)	10.5 (5–16)	0 0.0%	3 23.1%	10 76.9%
	2022 (n = 10)	10.8 (6–14)	0 0.0%	2 20%	8 80%
	2023 (n = 26)	11.0 (5.0–21)	0 0.0%	3 11.5%	23 88.5%
MF Mince	2021 (n =12)	6.3 (1–14)	0 0%	1 8.3%	11 91.7%
	2022 (n = 10)	4.7 (1–10)	0 0.0%	0 0.0%	10 100%
	2023 (n = 12)	5.2 (1–12)	0 0.0%	0 0.0%	12 100%
MF Chicken	2021 (n = 67)	7.6 (2–14)	3 4.5%	15 22.4%	49 73.1%
	2022 (n = 68)	7.6 (3–14)	3 4.4%	19 27.9%	46 67.6%
	2023 (n = 84)	7.0 (2–13)	1 1.2%	26 31%	57 67.9%
FF Seafood	2021 (n = 9)	7.8 (5–13)	4 44.4%	3 33.3%	2 22.2%
	2022 (n = 10)	7.3 (5–11)	5 50%	3 30%	2 20%
	2023 (n = 16)	6.6 (4–11)	11 68.8%	3 18.8%	2 12.5%
MF Beef	2021 (n = 13)	8.5 (5–20)	0 0.0%	0 0.0%	13 100%
	2022 (n = 8)	6.9 (4–9)	0 0.0%	0 0.0%	8 100%
	2023 (n = 12)	6.9 (4–10)	0 0.0%	0 0.0%	12 100%
MF Meatballs	2021 (n = 14)	8.6 (4–14)	0 0.0%	0 0.0%	14 100%
	2022 (n = 12)	8.3 (4–14)	0 0.0%	0 0.0%	12 100%
	2023 (n = 14)	7.4 (4–12)	0 0.0%	0 0.0%	14 100%
Tofu/Tempeh	2021 (n = 18)	4.7 (0–10)	0 0.0%	3 16.7%	15 83.3%
	2022 (n = 16)	3.7 (0–10)	0 0.0%	1 6.3%	15 93.8%
	2023 (n = 17)	3.3 (0–10)	0 0.0%	0 0.0%	17 100%
MF Ready meals	2021 (n = 42)	5.7 (1–13)	11 26.2%	21 50.0%	10 23.8%
	2022 (n = 44)	6.0 (1.0–13.0)	11 25.0%	24 54.5%	9 20.5%
	2023 (n = 46)	6.4 (1–13)	10 21.7%	17 63.0%	7 15.2%
Other	2021 (n = 19)	6.5 (0–11)	6 31.6%	2 10.5%	11 57.9%
	2022 (n = 15)	6.3 (0–12)	4 26.7%	3 20.0%	8 53.3%
	2023 (n = 18)	8.2 (0–19)	2 11.1%	5 27.8%	11 61.1%
Vegetable-based dishes	2021 (n = 9)	4 (3–7)	4 44.4%	5 55.6%	0 0.0%
	2022 (n = 7)	3.9 (3–6)	4 57.1%	3 42.9%	0 0.0%
	2023 (n = 7)	4.4 (3–10)	3 42.9%	4 57.1%	0 0.0%
Legume-based dishes	2021 (n = 15)	3.1 (1–6)	3 20.0%	12 80.0%	0 0.0%
	2022 (n = 13)	3.8 (1–11)	4 30.8%	9 69.2%	0 0.0%
	2023 (n = 16)	3.2 (1–6)	4 25.0%	12 75.0%	0 0.0%
MF Pizza	2021 (n = 4)	9.3 (5–13)	2 50.0%	2 50.0%	0 0.0%
	2022 (n = 6)	9.0 (5–12)	4 66.7%	2 33.3%	0 0.0%
	2023 (n = 8)	8.9 (5–12)	7 87.5%	1 12.5%	0 0.0%
MF Sauce	2021 (n = 5)	4.2 (2–6)	0 0.0%	0 0.0%	5 100%
	2022 (n = 3)	4.0 (2.0–6.0)	0 0.0%	0 0.0%	3 100%
	2023 (n = 2)	5.5 (5–6)	0 0.0%	0 0.0%	2 100%

* The A-score is a composite score derived from a sum of points allocated for the energy (kJ), saturated fat (g), total sugars (g) and sodium (mg) content per 100 g of food, with the number of points awarded dependent on thresholds set out in the UK’s nutrient profiling technical guidance document [49]. β Based on EU thresholds for protein claims whereby ≥12% to <20% energy from protein = ‘source of protein’ and ≥20% energy from protein = ‘high in protein’ claim [51].

**Table 2:** Micronutrient content (median and range) of categories with listed micronutrient contents on-pack across the three timepoints.

Category	Year N, %	Vitamin B12 μg/100 g	No. Products ‘High in’ Vitamin B12 * N, %	Year N, %	Iron mg/100 g	No. Products ‘High in’ Iron * N, %
MF Burgers	2021 2, 4%	0.38 ^1^	0 (0%)	2021 3, 6%	2.1 (2.1–4.1)	0 (0%)
	2022 3, 7%	0.8 (0.7–0.8)	2 (67%)	2022 3, 7%	3.1 (2.3–4.6)	1 (33%)
	2023 4, 7%	0.8 (0.7–1)	3 (75%)	2023 4, 7%	3.9 (2.3–4.9)	2 (50%)
MF Bacon/Slices	2021 4, 31%	0.8 (0.4–7.1)	2 (50%)	2021 4, 31%	3.3 (2.1–10)	1 (25%)
	2022 6, 60%	0.7 (0.4–7.1)	2 (33%)	2022 6, 60%	3.3 (2.1–10)	2 (33%)
	2023 8, 31%	0.7 (0.3–8.3)	3 (38%)	2023 7, 27%	3.8 (1.9–5.8)	3 (43%)
MF Chicken	2021 7, 10%	0.4 (0.4–0.7)	0 (0%)	2021 7, 10%	2.1 (2.1–4.0)	0 (0%)
	2022 6, 9%	0.6 (0.4–0.8)	1 (17%)	2022 6, 9%	2.6 (2.1–3.8)	0 (0%)
	2023 13, 15%	0.6 (0.4–1.4)	3 (23%)	2023 13, 15%	3.7 (2.1–6.1)	2 (15%)
MF Beef	2021 3, 23%	0.5 (0.4–2.4)	1 (33%)	2021 6, 46%	2.95 (2.1–4.2)	1 (17%)
	2022 2, 25%	- (0.5–2.4) ^2^	1 (50%)	2022 4, 50%	2.95 (2.5–3.3)	0 (0%)
	2023 5, 42%	0.8 (0.6–2.4)	3 (60%)	2023 5, 42%	4.7 (2.9–6.6)	3 (60%)
Other	2021 3, 16%	0.4 (0.4–1.1)	1 (33%)	-	-	-
	2023 4, 22%	0.8 (0.5–1.0)	3 (75%)	-	-	-

* Based on EU thresholds for nutrient claims for vitamin B12 (≥0.75 μg per 100 g) and iron (≥4.2 mg/100 mg per 100 g) [51]. ^1^ Two products containing 0.38 μg per 100 g; therefore, no range provided. ^2^ Only 2 products available; therefore, the median was not calculated and only the range is provided.

**Table 3:** Average (median and range) number of ingredients and number and percentage of products with ≥ 1 allergen * per category per timepoint.

Category	2021	n, (%) Products with ≥1 Allergen	2022	n, (%) Products with ≥1 Allergen	2023	n, (%) Products with ≥1 Allergen
*MF Burgers*	19.6 (4–37)	28 (57%)	20.3 (4–37)	23 (52%)	19.4 (2–35)	31 (55%)
*MF Sausages*	16.7 (7–33)	15 (36%)	17.3 (7–35)	12 (30%)	17.0 (2–34)	14 (31%)
*MF Pastry*	15.3 (2–29)	15 (79%)	16.2 (2–29)	12 (80%)	19.4 (2–32)	14 (74%)
*MF Bacon/slices*	12.1 (5–20)	4 (31%)	13.6 (5–24)	3 (30%)	16.4 (5–27)	13 (50%)
*MF Mince*	11.7 (6–23)	8 (67%)	10.8 (7–19)	6 (60%)	14.8 (7–27)	7 (58%)
*MF Chicken*	14.8 (2–31)	32 (48%)	17.0 (2–37)	35 (51%)	18.0 (2–37)	48 (57%)
*MF Seafood*	17.8 (10–24)	6 (67%)	20.5 (10–38)	4 (40%)	16.9 (7–38)	6 (38%)
*MF Beef*	14.0 (2–32)	7 (54%)	15.4 (4–32)	4 (50%)	13.5 (3–29)	7 (58%)
*MF Meatballs*	18.1 (8–30)	7 (50%)	19.6 (2–30)	6 (50%)	20.8 (8–31)	9 (64%)
*Tofu/tempeh*	5.9 (1–23)	12 (67%)	6 (1–24)	11 (69%)	5.8 (1–28)	12 (71%)
*MF Ready-meals*	19.9 (2–46)	13 (31%)	20.9 (2–46)	18 (41%)	19.9 (2–35)	18 (39%)
*Other*	18.2 (3–33)	9 (47%)	15.7 (3–27)	5 (33%)	17.6 (9–35)	5 (33%)
*Vegetable-based dishes*	24.7 (20–32)	2 (22%)	26.3 (20–32)	2 (29%)	21 (3–36)	1 (14%)
*Legume-based dishes*	29.3 (16–46)	4 (27%)	29.8 (17–45)	3 (23%)	22.1 (4–39)	7 (44%)
*MF Pizza*	19.3 (6–26)	3 (75%)	19.5 (8–26)	5 (83%)	20.0 (14–26)	8 (100%)
*MF Sauce*	9.4 (2–25)	2 (40%)	13.7 (5–25)	0 (0%)	13.5 (11–16)	1 (50%)

* Allergens were identified according to the 14 major allergens recognised by law in the EU [52]. MF: meat-free.

**Table 4:** Top 10 most frequently used ingredients and additives in all PBMAs at three timepoints.

2021		**2022**		**2023**	
Ingredients					
*Frequency*	*Description*	*Frequency*	*Description*	*Frequency*	*Description*
253	Salt	256	Salt	310	Salt
230	Water	230	Water	299	Water
185	Rapeseed Oil	181	Rapeseed Oil	237	Rapeseed Oil
120	Sunflower Oil	111	Sunflower Oil	133	Yeast extract
120	Onion	106	Yeast extract	118	Onion Powder
117	Yeast extract	105	Onion	117	Onion
112	Natural Flavourings	92	Onion Powder	114	Wheat Flour
89	Onion Powder	82	Black Pepper	104	Dextrose
84	Black Pepper	79	Wheat Flour	96	Natural Flavourings
83	Wheat Flour	78	Sugar	92	Sugar
**Additives**					
170	Methyl cellulose	101	Calcium carbonate	142	Methyl cellulose
167	Calcium carbonate	89	Methyl cellulose	83	Calcium carbonate
26	Calcium acetate	25	Sulphites	42	Plain caramel
16	Citric acid	14	Lactic acid	29	Citric acid
16	Carrageenan	11	Calcium acetate	28	Calcium acetate
15	Pectins	11	Citric acid	22	Sulphites
14	Sodium alginate	10	Carrageenan	21	Carrageenan
13	Sodium metabisulphite	8	Potassium lactate	20	Lactic acid
9	Potassium sorbate	7	Xanthan gum	15	Sodium alginate
9	Potassium chloride	7	Sodium alginate	13	Potassium lactate
		7	Caramelised sugar powder		

**Table 5:** Average (mean and SD and median and IQR) nutritional content of PBMAs (available in 2023) compared to meat products within the same category.

	Burgers					Sausages				
	PBMA (n = 56)		MB (n = 8)		*p* Value	PBMA (n = 45)		MB (n = 11)		*p* Value
	Mean (SD)	Median (IQR)	Mean (SD)	Median (IQR)		Mean (SD)	Median (IQR)	Mean (SD)	Median (IQR)	
Energy (kcal)	206.6 (40.5)	205.5 (176.0–235.3)	280.6 (40.5)	289.5 (238.3–318.8)	<0.001 ^1^	199.8	198.0 (155.0–221.0)	249.6	275.0 (160.0–308.0)	0.03 ^2^
Protein (g)	12.0	12.5 (5.8–17.0)	21.2	21.1 (16.2–26.1)	<0.001 ^2^	12.6 (3.8)	11.3 (9.6–16.0)	15.0 (2.0)	14.5 (13.8–16.2)	0.06 ^1^
Fat (g)	10.5 (4.4)	10.0 (7.1–14.0)	20.0 (4.5)	21.1 (15.3–24.3)	<0.001 ^1^	11.1	11.0 (6.2–13.2)	17.8	21.1 (6.1–23.9)	<0.01 ^2^
Saturates (g)	2.1	1.1 (0.7–2.1)	7.8	8.4 (5.3–10.7)	<0.001 ^2^	2.2	1.2 (1.0–3.6)	6.5	8.0 (2.2–8.5)	<0.001 ^2^
CHO (g)	14.0	13.4 (6.4–21.9)	4.3	1.1 (0.1–7.6)	0.001 ^2^	10.2	10.0 (5.7–14.0)	7.8	9.6 (2.6–10.7)	0.13 ^2^
Sugars (g)	1.8	1.4 (0.7–2.6)	1.0	0.9 (0.1–1.8)	0.09 ^2^	1.6	1.3 (0.6–2.5)	1.8	1.5 (0.9–2.8)	0.49 ^2^
Fibre (g)	4.2	4.8 (2.1–6.2)	0.2	0.7 (0.4–0.7)	<0.001^2^	4.6	4.6 (3.3–6.4)	2.0	4.6 (3.3–7.3)	<0.001 ^2^
Salt (g)	1.1 (0.3)	1.1 (0.9–1.3)	0.3 (0.1)	0.9 (0.4–1.1)	0.02^1^	1.4	1.3 (1.1–1.5)	1.5	1.4 (1.3–1.6)	0.07 ^2^
Total A points	7.8	7.5 (5.0–9.0)	13.4	12.0 (10.3–17.5)	<0.001^2^	8.8	8.0 (7.0–10.0)	14.8	17.0 (11.0–17.0)	<0.001^2^
	**Bacon/Slices**					**Mince**				
	**PBMA** **(n = 26)**		**MB** **(n = 20)**		***p*** **Value**	**PBMA** **(n = 12)**		**MB** **(n = 12)**		***p*** **Value**
	**Mean (SD)**	**Median (IQR)**	**Mean (SD)**	**Median (IQR)**		**Mean (SD)**	**Median (IQR)**	**Mean (SD)**	**Median (IQR)**	
Energy (kcal)	194.1	195.5 (143.8–223.3)	273.1	284.5 (221.5–311.5)	<0.001^2^	166.0 (67.5)	156.0 (95.3–235.0)	192.4 (42.5)	200.0 (145.3–221.0)	0.27 ^1^
Protein (g)	19.3	17.7 (14.0–25.3)	23.0	23.8 (19.3–24.7)	0.06^2^	18.7 (4.7)	19.0 (13.5–22.5)	21.1 (4.1)	21.4 (19.2–24.4)	0.19 ^1^
Fat (g)	9.1	7.4 (5.0–12.2)	20.0	21.1 (16.1-23.5)	<0.001^2^	6.7	3.5 (1.7–11.7)	11.9	12.8 (9.8–15.7)	0.04 ^2^
Saturates (g)	1.6	0.9 (0.6–1.5)	7.4	8.0 (6.1–8.7)	<0.001^2^	1.5	0.7 (0.4–2.6)	5.2	5.8 (3.6–6.8)	<0.001 ^2^
CHO (g)	6.3	5.8 (3.7–8.0)	0.2	0.0 (0.0–0.0)	<0.001^2^	5.5	5.7 (2.3–7.4)	0.2	0.0 (0.0–0.0)	<0.001 ^2^
Sugars (g)	1.7	1.3 (0.8–2.6)	0.2	0.0 (0.0–0.0)	<0.001^2^	1.0	0.7 (0.1–2.1)	0.1	0.0 (0.0–0.0)	<0.001 ^2^
Fibre (g)	5.6	5.3 (4.3–6.9)	0.0	0.0 (0.0–0.0)	<0.001^2^	4.8	5.4 (2.7–2.1)	0.0	0.0 (0.0–0.0)	<0.001 ^2^
Salt (g)	2.2 (0.8)	2.2 (1.5–2.8)	3.5 (0.7)	3.5 (2.9–4.1)	<0.001^1^	0.7	0.8 (0.2–0.9)	0.2	0.2 (0.2–0.2)	0.05 ^2^
Total A points	11.0 (3.8)	11.0 (8.0–13.0)	20.1 (3.5)	21.0 (18.3–22.5)	<0.001^1^	5.2 (3.6)	5.5 (1.5–7.5)	6.8 (2.8)	7.0 (5.0–8.0)	0.22 ^1^
	**Seafood**					**Beef**				
	**PBMA** **(n = 16)**		**MB** **(n = 14)**		***p*** **Value**	**PBMA** **(n = 12)**		**MB** **(n = 8)**		***p*** **Value**
	**Mean (SD)**	**Median (IQR)**	**Mean**	**Median (IQR)**		**Mean (SD)**	**Median (IQR)**	**Mean (SD)**	**Median (IQR)**	
Energy (kcal)	213.7	216.5 (185.3–245.0)	188.4	226.0 (99.5–240.0)	0.76 ^2^	162.0 (65.4)	150.5 (105.0–207.8)	177.6 (20.1)	184.0 (160.8–191.0)	0.52 ^1^
Protein (g)	5.9	4.8 (3.2–7.8)	17.5	16.8 (13.0–23.0)	<0.001 ^2^	18.0	15.5 (13.5–21.7)	26.7	28.4 (22.9–28.7)	0.004 ^2^
Fat (g)	10.4	9.7 (7.5–12.0)	8.6	10.5 (0.8–14.7)	0.82 ^2^	5.9 (3.9)	5.5 (2.8–9.6)	7.9 (1.0)	7.9 (7.2–8.7)	0.12 ^1^
Saturates (g)	1.4	1.2 (0.7–2.0)	2.0	1.4 (0.2–1.8)	0.95 ^2^	1.5 (1.4)	1.1 (0.6–2.0)	3.6 (0.5)	3.6 (3.3–3.9)	<0.001 ^1^
CHO (g)	22.2	24.3 (19.3–27.3)	12.4	15.1 (0.0–19.8)	<0.001 ^2^	6.8	6.4 (3.0–8.6)	0.0	0.0 (0.0–0.0)	<0.001 ^2^
Sugars (g)	2.6	2.1 (1.2–2.7)	0.9	1.0 (0.0–1.3)	0.01 ^2^	1.4	1.2 (0.5–2.1)	0.0	0.0 (0.0–0.0)	<0.001 ^2^
Fibre (g)	4.2 (3.9)	3.0 (2.3–4.2)	0.9 (0.8)	0.5 (0.0–1.8)	<0.001 ^2^	4.4 (2.2)	4.7 (2.7–5.4)	-	-	-
Salt (g)	0.9 (0.3)	0.9 (0.7–1.2)	0.6 (0.3)	0.4 (0.3–0.8)	<0.01 ^1^	1.1	1.1 (1.0–1.3)	0.2	0.2 (0.1–0.2)	<0.001 ^2^
Total A points	6.6 (1.9)	6.5 (5.0–8.0)	6.2 (3.5)	6.5 (2.0–9.3)	0.70 ^1^	6.9 (2.0)	6.5 (6.0–8.8)	14.8 (0.9)	15.0 (14.3–15.0)	<0.001 ^1^
	**Pastry/Pies**					**Chicken**				
	**PBMA(n = 19)**		**MB(n = 5)**		***p*** **Value**	**PBMA(n = 84)**		**MB(n = 4)**		***p*** **Value**
	**Mean (SD)**	**Median (IQR)**	**Mean (SD)**	**Median (IQR)**		**Mean (SD)**	**Median (IQR)**	**Mean (SD)**	**Median (IQR)**	
Energy (kcal)	290.1 (49.0)	288.0 (267.0–316.0)	336.4 (51.6)	352.0 (282.5–382.5)	0.08 ^1^	205.7 (57.6)	205.0 (164.8–252.8)	157.3	154.5 (147.3–170.0)	<0.001 ^1^
Protein (g)	9.5 (2.1)	9.6 (7.5–11.2)	8.4 (1.5)	8.4 (6.9-9.9)	0.29^1^	14.4	13.5 (11.0-17.1)	30.1	29.8 (29.1–31.5)	<0.001 ^2^
Fat (g)	15.1 (3.6)	14.7 (13.2–16.4)	23.2 (4.6)	24.1 (18.5–27.4)	<0.001 ^1^	9.8	9.5 (5.8–14.5)	4.1	3.9 (2.4–6.0)	0.01 ^2^
Saturates (g)	6.1	6.2 (5.2–6.7)	9.3	10.1 (7.8–10.5)	0.001 ^2^	1.4	1.2 (0.8–1.6)	0.8	0.8 (0.2–0.9)	0.03 ^2^
CHO (g)	27.6 (4.8)	29.0 (23.1–30.0)	26.0 (1.5)	25.7 (24.8–27.4)	0.49 ^1^	12.6	12.6 (4.7–19.1)	0.0	0.0 (0.0–0.0)	<0.001 ^2^
Sugars (g)	2.1 (0.6)	2.1 (1.8–2.6)	1.9 (0.4)	2.1 (1.4–2.2)	0.34 ^1^	1.4	1.0 (0.5–1.8)	0.0	0.0 (0.0–0.0)	<0.001 ^2^
Fibre (g)	3.0 (0.8)	3.0 (2.3–3.6)	3.0 (0.4)	2.9 (2.7–3.4)	0.96 ^1^	4.8	5.0 (3.3–6.4)	0.0	0.0 (0.0–0.0)	<0.001 ^2^
Salt (g)	1.0 (0.3)	1.0 (0.8–1.2)	1.0 (0.4)	1.2 (0.6–1.4)	0.82 ^1^	1.0	1.0 (0.8–1.3)	0.1	0.2 (0.1–0.2)	<0.001 ^2^
Total A points	12.7 (2.7)	13.0 (11.0–14.0)	20.6 (2.2)	20.0 (19.0–22.5)	<0.001^1^	7.0	7.0 (6.0-8.0)	4.0	1.5 (1.0–9.5)	0.10 ^2^

PBMAs—plant-based meat alternatives; MB—meat-based; SD—standard deviation; IQR—interquartile range. ^1^ Independent samples *t*-test used. ^2^ Mann-Whitney-U test used. CHOs—carbohydrates. Independent samples *t*-tests used to measure *p* value. *p* < 0.05 considered statistically significant.

## Data Availability

The original contributions presented in the study are included in the article, further inquiries can be directed to the corresponding author.

## Supplementary Materials

The following supporting information can be downloaded at: https://www.mdpi.com/article/10.3390/foods14050903/s1, Table S1: Category names and descriptions; Table S2: Brands available at each timepoint (number and percentage of products); Table S3: Average (median and range) energy and nutrient content (per 100g) of PBMAs for all categories and timepoints (2021, 2022 and 2023); Table S4: Frequency of main protein sources for products at each timepoint grouped according to % of energy from protein; Table S5: Nutrition claims made on products across the three timepoints and the proportion of products with nutrition claims and being ‘high’ in fat, saturated fat, sugars or salt; Table S6: Frequency (number and %) of lifestyle related claims at the three timepoints; Figure S1: Frequency (%) of PB and MB products per category with low, medium and high contents of fat, saturated fat sugars and salt; Figure S2: Proportion of PBMA and MB products per category with <12%, ≥12% to <20% and ≥20% energy from protein
