# Supplementary material for: Plant-Based Meat Alternatives on the Island of Ireland: Changes in the Market and Comparisons with Conventional Meat
Source: Foods. 2025 Mar 6;14(5):903. doi: 10.3390/foods14050903 (PMC11899366; doi:10.3390/foods14050903)
Supplement: Supplementary file 1 [file foods-14-00903-s001.zip › foods-3453012-supplementary.pdf]

Supplemental Table S1: Category names and descriptions

Category	Description
Meat free Burgers	All meat free burgers including meat free chicken, beef and bean burgers
Meat Free Sausages	Meat Free Sausages, anything that includes 'sausages', 'Chipolata' 'Vegfurter' 'Shroomdogs' or 'bangers'
Meat free Pastry products	E.g., sausage rolls, pasty etc
Meat free cured/sliced meats	Meat Free Bacon, Meat free Ham Slices, Vegan Pepperoni etc
Meat free Mince	Meat Free Mince, includes mince in name of food product, e.g. 'vegemince', 'No Beef Mince'
Meat free Chicken	Meat Free Chicken, anything trying to mimic Chicken such as 'Meat Free Chicken Nuggets' 'Meat Free Chicken Strips'
Fish Free Seafood	Meat Free Seafood, e.g. 'Fishless Fish Fingers', 'Fish Free Cakes', 'Fishless scampi'
Meat free Beef	Meat Free Beef Product, e.g. 'Steaks', 'No Beef Strips'
Meat free Meat balls	Meat free meatballs
Tofu/Tempeh	Foods including Tofu or tempeh as a main ingredient, 'Teriyaki Block Tofu'
Meals	Ready-meals and products which can be eaten alone eg., cottage pie or lasagne
Other	Other meat free products that don't fall under the other titles e.g. 'Quorn Family Roast', 'Meat Free Picnic Egg', 'Nut Cutlets'
Vegetable Based Dishes	Vegetable Rich Ready Made Meals, anything not trying to mimic meat changing the recipe to be vegetable based e.g. 'Sweet Potato Katsu Curry' 'Mushroom Bolognese'
Legume Based Dishes	Legume Rich Ready Made Meals, anything not trying to mimic meat changing the recipe to be Legume based e.g. 'Lentil Lasagne'
Meat free sauce	e.g., meat free bolognese or no chicken curry
Meat free pizza	Pizza which contains meat substitute toppings e.g 'Vegan Pepperoni Pizza'

Supplemental Table S2: Brands available at each timepoint (number and percentage of products)

2021			2022			2023		
**Brand (n=47)**	**Number**	**% of products**	**Brand (n=52)**	**Number**	**% of products**	**Brand (n=61)**	**Number**	**% of products**
Quorn	54	15	Tesco Plant Chef	44	14	Quorn	70	18
Sainsbury's Plant Pioneers	40	11	Quorn	34	11	Tesco Plant Chef	46	12
Tesco Plant Chef	37	11	Sainsbury's Plant Pioneers	33	10	Sainsbury's Plant Pioneers	41	10
Linda McCartney Foods	21	6	Linda McCartney Foods	22	7	Linda McCartney Foods	23	6
Fry's Family Foods UK (Pty) Ltd	16	5	Wicked Kitchen	17	5	Squeaky Bean	19	5
Birds Eye Limited	13	4	Birds Eye Limited	13	4	Richmond	16	4
Vivera B.V.,	13	4	Sainsbury's Love Your Veg!	11	3	Birds Eye Limited	14	4
Wicked Kitchen	13	4	Fry's Family Foods UK (Pty) Ltd	10	3	Beyond Meat	13	3

Sainsbury's Love Your Veg!	12	3	The Tofoo Company Ltd	8	2	This Isn't	12	3
Naked Glory	11	3	Cauldron	7	2	Vivera B.V.,	12	3
Squeaky Bean	10	3	Heck! Food Ltd	7	2	Wicked Kitchen	11	3
The Tofoo Company Ltd	10	3	Squeaky Bean	7	2	Cauldron	8	2
Heck! Food Ltd	8	2	This Isn't	8	2	Meatless Farm	7	2
Cauldron	5	1	Vivera B.V.,	7	2	The Tofoo Company Ltd	7	2
This Isn't	4	1	Beyond Meat	6	2	Fry's Family Foods UK (Pty) Ltd	6	2
Meatless Farm	4	1	Richmond	6	2	Oumph!	6	2
The Vegetarian Butcher	4	1	The Vegetarian Butcher	6	2	The Vegetarian Butcher	6	2
Richmond	4	1	VFC Foods Ltd	5	2	Chicago Town	5	1
Future Farm	4	1	Finnebrogue Artisan	4	1	Sainsbury's Supermarket Ltd	5	1
Oumph!	4	1	Meatless Farm	4	1	VFC Foods Ltd	5	1
LikeMeat GmbH	4	1	Naked Glory	4	1	Strong Roots	4	1
Finnebrogue Artisan	4	1	Oumph!	4	1	Dopsu	3	1
Tofurky	4	1	Strong Roots	4	1	Planted Foods GmbH	3	1

Tesco Stores Ltd	4	1	Tesco Stores Ltd	4	1	Sainsbury's Taste the difference	3	1
Sainsbury's Supermarket Ltd	4	1	Chicago Town	3	1	Zizzi	3	1
GoodLife Foods	4	1	GoodLife Foods	3	1	Fridge Raiders	2	1
LEON Grocery Ltd	4	1	Sainsbury's Supermarket Ltd	3	1	Future Farm	2	1
Moving Mountains Foods Ltd	3	1	Biff's Kitchen Ltd	2	1	Gosh!	2	1
Beyond Meat	3	1	Curators of Flavour Ltd	2	1	Happiee!	2	1
Strong Roots	3	1	Dopsu	2	1	Itsu	2	1
Plant Power	3	1	Future Farm	2	1	Moving Mountains Foods Ltd	2	1
Lazy Vegan BV	3	1	Itsu	2	1	Paxo	2	1
Amy's Kitchen Uk Ltd	3	1	Lazy Vegan BV	2	1	Peperami	2	1
Chicago Town	2	1	LikeMeat GmbH	2	1	Pieminister	2	1
Pukka Pies Ltd	2	1	Marlow Foods Ltd	2	1	Pukka Pies Ltd	3	1

Curators of Flavour Ltd	2	1	Moving Mountains Foods Ltd	2	1	Rustlers	2	1
Naturli' Foods A/S	1	0	Plant Power	2	1	Tesco Stores Ltd	2	1
Scratch Meals Ltd	1	0	Pukka Pies Ltd	2	1	White rabbit	2	1
Clearspring Ltd	1	0	Zizzi	2	1	Aunt Bessie's	1	0
Hiltons Foods Ltd	1	0	Aunt Bessie's	1	0	Better Nature	1	0
Higgidy Ltd	1	0	Bighams	1	0	Biff's Kitchen Ltd	1	0
Rustlers	1	0	BOL	1	0	Bighams	1	0
Kings Elite Snacks,	1	0	Clearspring Ltd	1	0	Clearspring Ltd	1	0
Bighams	1	0	Garden Gourmet	1	0	Curators of Flavour Ltd	1	0
Aunt Bessie's	1	0	Goodfellas	1	0	Dr. Oetker	1	0
Itsu	1	0	Gosh!	1	0	Finnebrogue Artisan	1	0
BOL	1	0	Higgidy Ltd	1	0	Ginsters	1	0
			Hiltons Foods Ltd	1	0	Goodfellas	1	0
			LEON Grocery Ltd	1	0	GoodLife Foods	1	0
			Rustlers	1	0	Heck! Food Ltd	1	0
			Shicken Foods	1	0	Higgidy Ltd	1	0

			The Meatless Farm	1	0	Hiltons Foods Ltd	1	0
						Kirsty's	1	0
						La Vie	1	0
						Mori-Nu	1	0
						Plant Power	1	0
						Princes	1	0
						Sainsbury's Free From	1	0
						Shicken Foods	1	0
						The Meatless Farm	1	0
						Wall's	1	0

Supplemental Table S3: Average (median and range) energy and nutrient content (per 100g) of PBMAs for all categories and timepoints (2021, 2022 and 2023)

	Burgers				Sausages				Pastry			
Year	2021	2022	2023	Change	2021	2022	2023	Change	2021	2022	2023	Change
N	49	44	56	↑	42	40	45	↑	19	15	19	/
Energy (kcal)	197 (107-298)	195 (107-298)	205.4 (134-305)	↑	163 (89-288)	183.5 (89-288)	198 (123-444)	↑	279 (178-377)	287 (178-377)	288 (178-377)	↑
Protein (g)	11.3 (3-21)	11.2 (3-19.8)	12.5 (3.4-21)	↑	11.9 (4.1-24)	11.5 (4.1-23.1)	11.3 (6.4-23.1)	↓	9.4 (5.6-12.1)	10.6 (6.1-12.6)	9.6 (6.3-12.6)	↑
Fat (g)	9.1 (1.8-21.1)	8.7 (1.8-21.1)	9.95 (2.7-20)	↑	8.6 (1.1-22.9)	10.5 (1.1-16)	11 (3.2-38)	↑	14.4 (7.6-21.5)	14.4 (7.6-21.5)	14.7 (7.6-21.5)	↑
Saturated fat (g)	1.1 (0.2-18)	1.1 (0.2-18)	1.1 (0.2-18)	/	1.2 (0.2-13.1)	1 (0.3-13.1)	1.2 (0.4-13.1)	/	6.3 (0.7-9.9)	6.3 (0.7-9)	6.2 (0.7-9.4)	↓
CHO (g)	11.9 (2.8-30.8)	11.7 (1.7-30.8)	13.4 (2.8-28.7)	↑	10.1 (2.2-21)	9.4 (1.8-21)	10 (2.2-25.2)	↓	27.2 (17-37.2)	27.2 (17-37.2)	29 (17-37.2)	↑
Sugars (g)	1.4 (0-8.1)	1.4 (0-8.1)	1.4 (0-6.3)	/	1.3 (0-5.1)	1.3 (0-5.1)	1.3 (0.1-4.6)	/	2.1 (0.7-5.5)	2.1 (0.6-3.4)	2.1 (0.6-3.4)	/
Fibre (g)	5 (0-10.4)	5.4 (0-10.4)	5 (0-7.6)	/	4.95 (0-10.2)	5.1 (0-10.2)	4.6 (0-10)	↓	3 (1.3-6.2)	3 (1.3-4.6)	3 (1.3-4.6)	/
Salt (g)	1.2 (0.1-2.1)	1.1 (0.1-1.8)	1.1 (0.5-2)	↓	1.3 (0.9-2.7)	1.3 (0.6-1.9)	1.3 (0.6-5.1)	/	1.1 (0.6-1.8)	0.98 (0.7-1.8)	0.98 (0.7-1.7)	↓
A-score	8.4 (4-19)	8.0 (4-19)	7.8 (5-18)	↓	9.1 (5-17)	8.6 (5-17)	8.8 (5-18)	↓	13.6 (9-17)	12.7 (5-17)	12.7 (5-18)	↓

	Bacon/slices				Mince				Chicken			
Year	2021	2022	2023	Change	2021	2022	2023	Change	2021	2022	2023	Change
N	13	10	26	↑	12	10	12	/	67	68	84	↑

Energy (kcal)	193 (95-347)	182 (87-347)	195.5 (92-399)	↑	153 (92-223)	133.5 (92-238)	156 (92-278)	↑	209.0 (84.0-321.0)	219.5 (94-318)	205.0 (84.0-318.0)	↓
Protein (g)	17 (10-31)	16.7 (10-31)	17.7 (10-31)	↑	18.3 (10-24)	19.95 (13-25.7)	18.95 (13-25.7)	↑	14.0 (2.1-32.0)	14.0 (2.1-32.0)	13.5 (3.0-27.0)	↓
Fat (g)	6.2 (0.5-21.1)	5.9 (0.6-21.2)	7.4 (0.5-29.4)	↑	3.5 (0.5-13)	2.2 (0.5-17)	3.5 (0.6-17)	/	8.3 (1.6-22.0)	9.5 (2.3-17.3)	9.5 (1.4-18.0)	↑
Saturated fat (g)	0.8 (0.1-7.1)	0.7 (0.1-6.1)	0.9 (0.1-8.5)	↑	0.8 (0.1-11)	0.5 (0.1-5.7)	0.7 (0.1-5.7)	↓	1.0 (0.3-6.1)	1.1 (0.3-6.1)	1.2 (0.4-6.1)	↑
CHO (g)	5.9 (1.7-12.7)	6.2 (1.4-14)	5.8 (1.7-14)	↓	7.9 (2.3-14)	6.5 (2.3-11.7)	5.7 (1.9-11.7)	↓	14.0 (1.4-27.4)	14.9 (1.7-27)	12.6 (1.1-27.0)	↓
Sugars (g)	1.2 (0.3-4.3)	1.2 (0.3-3.8)	1.3 (0.3-4.3)	↑	0.9 (0.1-3.2)	0.7 (0-3.2)	0.7 (0-3.2)	↓	1.0 (0.1-8.8)	1.3 (0.1-8.8)	1.0 (0.0-6.9)	/
Fibre (g)	5.4 (0-9.3)	5.8 (4-8.9)	5.4 (2-9.3)	/	5.1 (1.4-7.5)	5.4 (0-7.5)	5.4 (0-7.5)	↑	4.6 (0.0-12.0)	4.4 (0.0-8.4)	4.95 (0.0-8.4)	↑
Salt (g)	1.8 (0.9-3.5)	2.2 (1.1-3.5)	2.2 (0.9-3.8)	↑	0.5 (0.1-1.5)	0.8 (0.1-1.3)	0.8 (0.1-1.1)	↑	1.2 (0.4-2.8)	1.2 (0.4-2.8)	1.0 (0.0-1.9)	↓
A-score	10.5 (5-16)	10.8 (6-14)	11.0 (5.0-21)	↑	6.3 (1-14)	4.7 (1-10)	5.2 (1-12)	↓	7.6 (2-14)	7.6 (3-14)	7.0 (2-13)	↓

	**Seafood**				**Beef**				**Meatballs**			
Year	2021	2022	2023	Change	2021	2022	2023	Change	2021	2022	2023	Change
N	9	10	16	↑	13	8	12	↓	14	12	14	/
Energy (kcal)	214.0 (188.0-403.0)	227.0 (176.0-369.0)	216.5 (68.0-369.0)	↑	170.0 (120.0-320.0)	161.5 (105.0-222.0)	150.5 (73.0-276.0)	↓	177.0 (107.0-236.0)	181.5 (107.0-249.0)	171.0 (107.0-249.0)	↓
Protein (g)	8.3 (3.4-16.9)	6.7 (3.0-16.9)	4.8 (0.3-15.0)	↓	15.0 (13.0-30.0)	15.0 (13.0-30.0)	15.5 (12.8-34.4)	↑	15.1 (5.8-17.5)	15.2 (5.8-16.0)	15.2 (5.8-20.9)	↑
Fat (g)	9.3 (7.8-3-.5)	11.7 (7.7-25.5)	9.7	↑	6.4	6.1 (1.4-9.7)	5.5	↓	7.9 (3.1-15.0)	8.4 (5.5-18.0)	6.6 (5.3-18.0)	↓

			(1.0-25.5)		(0.7-10.0)		(0.4-12.4)					
Saturated fat (g)	1.0 (0.6-5.5)	1.5 (0.6-4.1)	1.2 (0.3-4.1)	↑	1.1 (0.1-4.7)	1.0 (0.4-2.3)	1.1 (0.1-4.7)	/	1.4 (0.6-9.0)	1.4 (0.5-5.2)	0.95 (0.5-5.2)	↓
CHO (g)	24.0 (18.7-29.0)	22.8 (8.9-27.4)	24.3 (7.9-29.6)	↑	7.5 (2.1-38.2)	8.2 (1.1-16.4)	6.4 (1.1-15.5)	↓	7.7 (5.8-16.3)	8.2 (4.4-16.3)	8.2 (4.4-15.0)	↑
Sugars (g)	1.6 (0.6-3.8)	1.8 (0.6-6.5)	2.1 (0.5-8.4)	↑	2.0 (0.3-22.7)	1.9 (0.2-9.0)	1.2 (0.2-3.8)	↓	1.1 (0.1-3.0)	1.5 (0.1-8.6)	1.3 (0.1-8.6)	↑
Fibre (g)	4.0 (2.5-11.0)	3.3 (2.0-18.0)	3.0 (1.9-18.0)	↓	4.9 (0.3-6.8)	5.0 (2.0-7.7)	4.7 (0.8-7.7)	↓	4.7 (1.0-7.1)	3.9 (0.0-7.1)	3.8 (0.0-7.1)	↓
Salt (g)	1.1 (0.7-1.6)	0.9 (0.5-1.3)	0.9 (0.5-1.4)	↓	1.2 (0.6-3.0)	1.1 (0.7-1.7)	1.1 (0.7-1.6)	↓	1.3 (0.6-1.9)	1.1 (0.8-1.9)	1.1 (0.8-1.5)	↓
A-score	7.8 (5-13)	7.3 (5-11)	6.6 (4-11)	↓	8.5 (5-20)	6.9 (4-9)	6.9 (4-10)	↓	8.6 (4-14)	8.3 (4-14)	7.4 (4-12)	↓

	**Tofu/tempeh**				**Meals**				**Other**			
Year	2021	2022	2023	Change	2021	2022	2023	Change	2021	2022	2023	Change
N	18	16	17	↓	42	44	46	↑	19	15	18	↓
Energy (kcal)	182.5 (64.0-316.0)	170.0 (64.0-239.0)	146.0 (56.0-232.0)	↓	136.5 (66.0-287.0)	141.0 (66.0-287.0)	155.0 (68.0-287.0)	↑	190.0 (20.0-304.0)	183.0 (20.0-256.0)	174.0 (20.0-278.0)	↓
Protein (g)	12.9 (6.8-21.3)	12.9 (6.8-21.3)	15.6 (6.5-21.3)	↑	5.6 (2.2-16.9)	5.7 (2.7-16.9)	5.9 (2.7-16.6)	↑	13.6 (1.0-27.0)	9.8 (1.0-22.4)	13.0 (1.0-22.1)	↓
Fat (g)	8.1 (3.0-17.0)	8.6 (3.0-17.0)	7.8 (2.6-17.0)	↓	4.2 (0.4-14.3)	4.7 (0.4-17.2)	5.5 (0.7-17.2)	↑	6.9 (0.3-18.3)	7.7 (0.3-20.0)	7.5 (0.3-20.0)	↑

Saturated fat (g)	1.3 (0.6-2.9)	1.3 (0.6-2.9)	1.2 (0.4-2.9)	↓	1.2 (0.0-6.6)	1.5 (0.0-7.6)	1.5 (0.1-7.6)	↑	0.7 (0.1-3.5)	1.0 (0.1-3.5)	1.0 (0.1-4.3)	↑
CHO (g)	2.9 (1.0-31.8)	2.5 (1.0-19.4)	1.8 (0.6-7.2)	↓	18.7 (5.0-34.8)	19.8 (4.3-34.8)	22.5 (4.3-34.8)	↑	8.9 (1.0-30.0)	9.2 (1.0-30.0)	9.2 (1.0-30.0)	↑
Sugars (g)	1.2 (0.1-11.0)	0.9 (0.1-2.3)	0.6 (0.1-5.9)	↓	2.5 (0.3-12.0)	2.6 (0.5-12.0)	2.6 (1.0-12.0)	↑	2.4 (0.3-18.9)	1.9 (0.3-8.2)	1.3 (0.0-16.0)	↓
Fibre (g)	2.5 (0.0-8.8)	1.5 (0.0-8.8)	1.3 (0.0-6.6)	↓	2.3 (1.4-5.1)	2.4 (1.0-4.6)	2.3 (0.0-5.6)	/	3.9 (0.0-9.1)	4.0 (0.0-9.1)	4.2 (0.0-5.0)	↑
Salt (g)	0.4 (0.0-1.5)	0.3 (0.0-1.5)	0.1 (0.0-1.5)	↓	0.6 (0.1-1.6)	0.6 (0.1-1.6)	0.6 (0.3-1.6)	/	0.9 (0.0-1.7)	0.9 (0.0-1.9)	1.3 (0.0-2.0)	↑
A-score	4.7 (0-10)	3.7 (0-10)	3.3 (0-10)	↓	5.7 (1-13)	6.0 (1.0-13.0)	6.4 (1-13)	↑	6.5 (0-11)	6.3 (0-12)	8.2 (0-19)	↑

	Veg-based dishes				Legume-based dishes				Pizza			
Year	2021	2022	2023	Change	2021	2022	2023	Change	2021	2022	2023	Change
N	9	7	7	↓	15	13	16	↑	4	6	8	↑
Energy (kcal)	114.0 (86.0-223.0)	139.0 (90.0-208.0)	114.0 (83.0-229.0)	/	109.0 (62.0-195.0)	98.0 (62.0-195.0)	105.0 (62.0-176.0)	↓	252.5 (145.0-266.0)	222.5 (174.0-266.0)	226.5 (174.0-266.0)	↓
Protein (g)	3.0 (2.2-5.7)	3.1 (2.2-5.1)	3.1 (2.0-6.0)	↑	3.7 (2.6-5.8)	3.6 (2.6-5.6)	3.5 (2.6-6.4)	↓	6.3 (4.4-10.0)	6.3 (4.9-10.0)	5.9 (4.7-6.8)	↓
Fat (g)	3.2 (2.7-12.9)	5.2 (2.4-9.7)	3.1 (2.4-9.5)	↓	2.8 (0.0-6.2)	2.2 (0.0-6.2)	2.2 (0.0-8.3)	↓	7.5 (4.6-11.0)	6.0 (3.1-11.0)	6.4 (5.1-11.0)	↓
Saturated fat (g)	1.1 (0.4-2.5)	1.0 (0.4-1.4)	1.0 (0.3-4.4)	↓	0.8 (0.0-2.5)	0.9 (0.0-2.5)	0.9 (0.0-2.1)	↑	3.4 (2.8-5.1)	3.4 (0.5-4.0)	3.4 (0.5-4.6)	/
CHO (g)	16.6 (11.5-26.3)	16.6 (11.5-26.3)	16.2 (10.2-28.2)	↓	13.4 (10.1-27.7)	13.6 (9.1-27.7)	15.3 (9.1-23.2)	↑	35.5 (17.0-38.0)	35.0 (24.0-37.0)	35.0 (24.0-37.0)	↓

Sugars (g)	3.4 (2.1-5.3)	3.1 (2.1-5.3)	2.9 (2.6-6.0)	↓	2.5 (1.8-8.3)	2.6 (1.8-8.3)	3.0 (1.7-4.8)	↓	3.5 (3.0-5.4)	2.9 (2.2-4.9)	2.9 (2.0-4.9)	↓
Fibre (g)	2.2 (1.1-3.4)	2.7 (1.1-3.7)	1.8 (1.5-3.4)	↓	3.2 (0.0-4.5)	2.9 (0.0-4.2)	2.7 (1.0-6.6)	↓	2.0 (1.7-3.4)	2.0 (1.7-3.0)	2.0 (1.7-3.0)	/
Salt (g)	0.5 (0.2-0.9)	0.5 (0.2-1.0)	0.5 (0.4-1.0)	/	0.5 (0.3-0.8)	0.5 (0.2-0.8)	0.5 (0.2-1.0)	/	1.0 (0.6-1.1)	1.0 (0.8-1.7)	0.9 (0.7-1.7)	↓
A-score	4 (3-7)	3.9 (3-6)	4.4 (3-10)	↑	3.1 (1-6)	3.8 (1-11)	3.2 (1-6)	↑	9.3 (5-13)	9.0 (5-12)	8.9 (5-12)	↓

	**Sauce**			
Year	2021	2022	2023	Change
N	5	3	2	↓
Energy (kcal)	94.0 (55.0-219.0)	94.0 (55.0-219.0)	175.0 (131.0-219.0)	↑
Protein (g)	6.4 (3.3-17.0)	6.4 (3.3-12.8)	12.9 (12.8-13.0)	↑
Fat (g)	3.1 (0.9-13.4)	3.1 (0.9-13.4)	8.6 (3.7-13.4)	↑
Saturated fat (g)	0.4 (0.1-1.9)	0.4 (0.1-1.0)	1.0 (0.9-1.0)	↑
CHO (g)	8.9 (6.2-9.4)	8.9 (6.8-9.0)	9.4 (9.0-9.7)	↑
Sugars (g)	2.8 (2.3-4.2)	2.7 (2.3-4.2)	3.1 (2.3-3.8)	↑
Fibre (g)	2.4 (0.8-5.7)	3.1 (2.4-5.7)	-	↑
Salt (g)	0.7 (0.6-1.0)	0.7 (0.6-1.0)	1.0 (1.0-1.0)	↑

A-score	4.2 (2-6)	4.0 (2.0-6.0)	5.5 (5-6)	↑

Supplemental Table S4: Frequency of main protein sources for products at each timepoint grouped according to % of energy from protein

	<12% energy from protein					
	2021 (n=56)		2022 (n=56)		2023 (n=63)	
	n	%	n	%	n	%
Vegetable	11	20	12	21	13	21
Mushroom	8	14	4	7	3	5
Jackfruit	7	13	7	13	8	13
Pea	7	13	7	13	5	8
Veg and legumes	6	11	8	14	7	11
Mycoprotein	5	9	1	2	4	6
Soy	3	5	5	9	4	6
Bean	3	5	4	7	5	8
Soy and wheat	2	4	1	2	0	0
Wheat	1	2	1	2	0	0
Other	1	2	2	4	5	8

Wheat and pea	0	0	0	0	1	2
Pea combinations	0	0	0	0	1	2

	≥12% energy from protein					
	2021 (n=92)		2022 (n=90)		2023 (n=104)	
	n	%	n	%	n	%
Soy	16	17	20	22	16	15
Mycoprotein	13	14	11	12	17	16
Soy and wheat	11	12	10	11	10	10
Veg and legumes	9	10	7	8	7	7
Pea	8	9	16	18	16	15
Wheat	6	7	6	7	10	10
Vegetable	6	7	4	4	3	3
Bean	6	7	3	3	5	5
Mushroom	4	4	4	4	3	3
Jackfruit	3	3	2	2	2	2
Wheat and pea	3	3	1	1	4	4
Tofu/tempeh	3	3	2	2	2	2
Soy combinations	2	2	0	0	0	0
Soy and pea	0	0	1	1	0	0
Other	0	0	1	1	2	2
Pea combinations	0	0	0	0	5	5
Wheat combinations	0	0	0	0	1	1

	≥20% energy from protein					
	2021 (n=202)		2022 (n=175)		2023 (n=231)	

	n	%	n	%	n	%
Soy	49	24	50	29	58	25
Soy and wheat	36	18	28	16	33	14
Mycoprotein	36	18	24	14	50	22
Pea	31	15	27	15	33	14
Wheat	16	8	8	5	10	4
Tofu/tempeh	11	5	11	6	12	5
Wheat and pea	7	3	12	7	16	7
Mushroom	5	2	5	3	2	1
Soy and pea	5	2	5	3	10	4
Soy combinations	4	2	3	2	4	2
Jackfruit	1	0	0	0	0	0
Pea combinations	1	0	2	1	0	0
Wheat combinations	0	0	0	0	3	1

Supplemental Table S5: Nutrition claims made on products across the three timepoints and the proportion of products with nutrition claims and being 'high' in fat, saturated fat, sugars or salt*

	2021				2022				2023			
	Total		Products with ≥1 'red' TLS		Total		Products with ≥1 'red' TLS		Total		Products with ≥1 'red' TLS	
Nutrition claims	n	%	n	%	n	%	n	%	n	%	n	%
High/Source of protein	169	89	36	21	151	87	35	23	216	89	38	18
High/Source of Fibre	88	46	15	17	77	45	13	17	90	37	9	10
Low in saturated fat	53	28	8	15	52	30	4	8	81	33	5	6
Source of Iron	21	11	3	14	15	9	3	20	18	7	3	17

Source of B12	15	8	2	13	14	8	2	14	18	7	3	18
Fortified with B12	6	3	2	33	6	3	2	33	13	5	2	15
Fortified with Iron	9	5	4	44	8	5	3	38	11	5	1	9
Low in Fat	18	9	0	0	10	6	4	40	8	3	3	38
Source of Omega 3	1	1	0	0	2	1	0	0	6	2	2	33
Rich in Vitamin A	3	2	0	0	3	2	0	0	4	2	0	0
Source of calcium	1	1	0	0	3	2	0	0	3	1	0	0
Low Sugar	3	2	0	0	3	2	0	0	4	2	0	0
Low salt	3	2	1	33	1	1	0	0	2	1	0	0
No added sugar	0	0	0	0	1	1	0	0	2	1	0	0
x% less saturated fat than meat	0	0	0	0	0	0	0	0	2	1	0	0
Source of Folic Acid	2	1	1	50	2	1	1	50	1	0	0	0
Fat free	1	1	0	0	1	1	0	0	1	0	0	0
source of magnesium	0	0	0	0	0	0	0	0	1	0	0	0
high in omega-3	0	0	0	0	0	0	0	0	1	0	0	0
Source of phosphorus	0	0	0	0	0	0	0	0	1	0	0	0
Source of Zinc	2	1	1	50	2	1	1	50	0	0	0	0
Total claims	190	54	40	21	173	54	38	22	243	61	40	16

*Based on thresholds from the UK's Front of Pack traffic light labelling system, whereby total fat, saturated fat, sugar and salt content was considered 'high' if it was >17.5g/100g, >5g/100g, >22.5g/100g, >1.5g/100g respectively (240).

Supplemental Table S6: Frequency (number and %) of lifestyle related claims at the three timepoints

	2021		2022		2023	
**Other Label Description**	n	%	n	%	n	%
Vegan friendly/suitable for vegans	183	58	206	68	288	75
Suitable for Vegetarians	149	47	117	39	140	37
Gluten free	55	17	41	14	46	12
100% Plant based/plant-based	46	14	49	16	77	20
No soy	33	10	28	9	43	11
Made with non GM ingredients	24	8	16	5	9	2
No artificial colours	20	6	14	5	13	3
Organic	18	6	11	4	13	3
No artificial flavours	15	5	10	3	12	3
No artificial Preservatives	13	4	11	4	14	4
Portion = 1 of your five a day	10	3	4	1	5	1
Kosher-PAREV	10	3	2	1	5	1
Halal	7	2	7	2	18	5
GM free	7	2	6	2	12	3
GMO Free	7	2	5	2	4	1
Antibiotics free	7	2	2	1	0	0
No Artificial Additives	6	2	8	3	5	1
No milk or lactose	2	1	1	0	0	0
Kosher-KLBD	1	0	4	1	4	1
All Natural Ingredients	1	0	5	2	4	1
palm oil free	1	0	2	1	3	1
5 of your 5 a day	1	0	1	0	1	0
Packed with real fruit/veg	1	0	1	0	0	0
suitable for coeliacs	1	0	0	0	0	0
Free from nitrites	1	0	1	0	0	0
ready to eat	0	0	2	1	17	4
Sustainable nutrition	0	0	0	0	14	4
2 of your 5 a day	0	0	1	0	4	1
No nuts or seeds	0	0	0	0	3	1
from soya protein	0	0	4	1	2	1
dairy free	0	0	0	0	2	1
No junk	0	0	0	0	1	0
Allergen-free	0	0	0	0	1	0
no fishing	0	0	1	0	1	0
ready to season	0	0	0	0	1	0
Sustainable soy beans	0	0	1	0	1	0

3 of your 5 a day	0	0	1	0	0	0

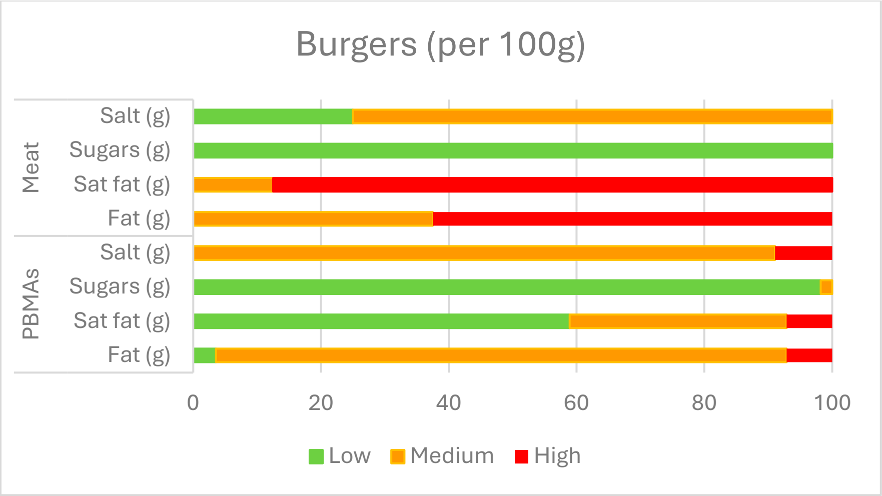
Burgers (per 100g)
Meat
PBMAs
Salt (g)
Sugars (g)
Sat fat (g)
Fat (g)
0
20
40
60
80
100
Low
Medium
High

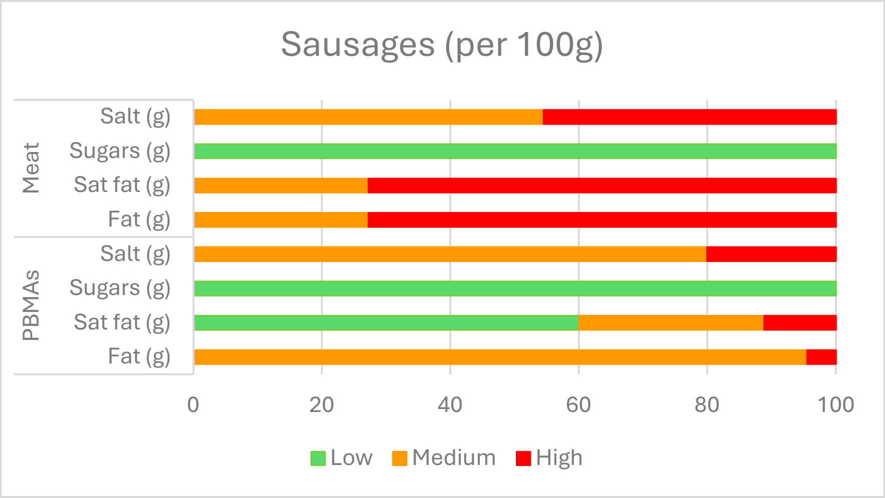
Sausages (per 100g)
Meat
PBMAs
Salt (g)
Sugars (g)
Sat fat (g)
Fat (g)
0
20
40
60
80
100
Low
Medium
High

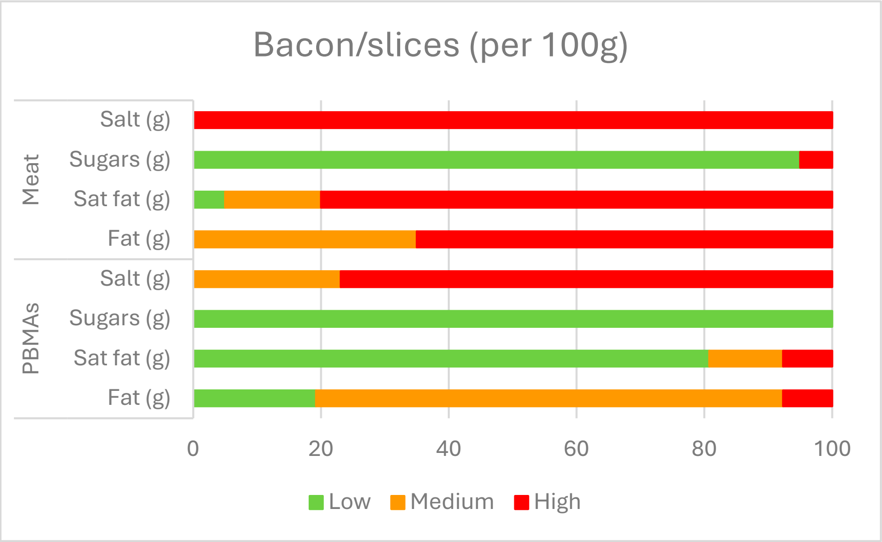
Bacon/slices (per 100g)
Meat
PBMAs
Salt (g)
Sugars (g)
Sat fat (g)
Fat (g)
0
20
40
60
80
100
Low
Medium
High

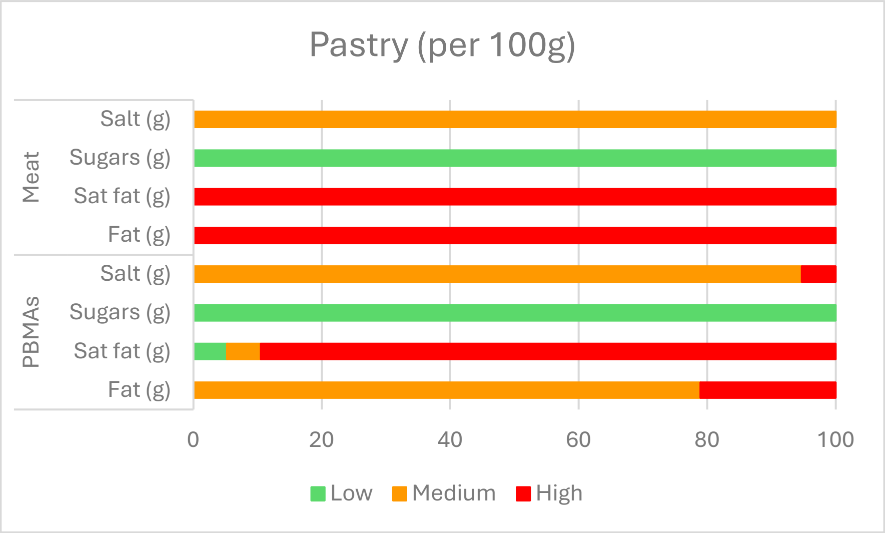
Pastry (per 100g)
Meat
PBMAs
Salt (g)
Sugars (g)
Sat fat (g)
Fat (g)
0
20
40
60
80
100
Low
Medium
High

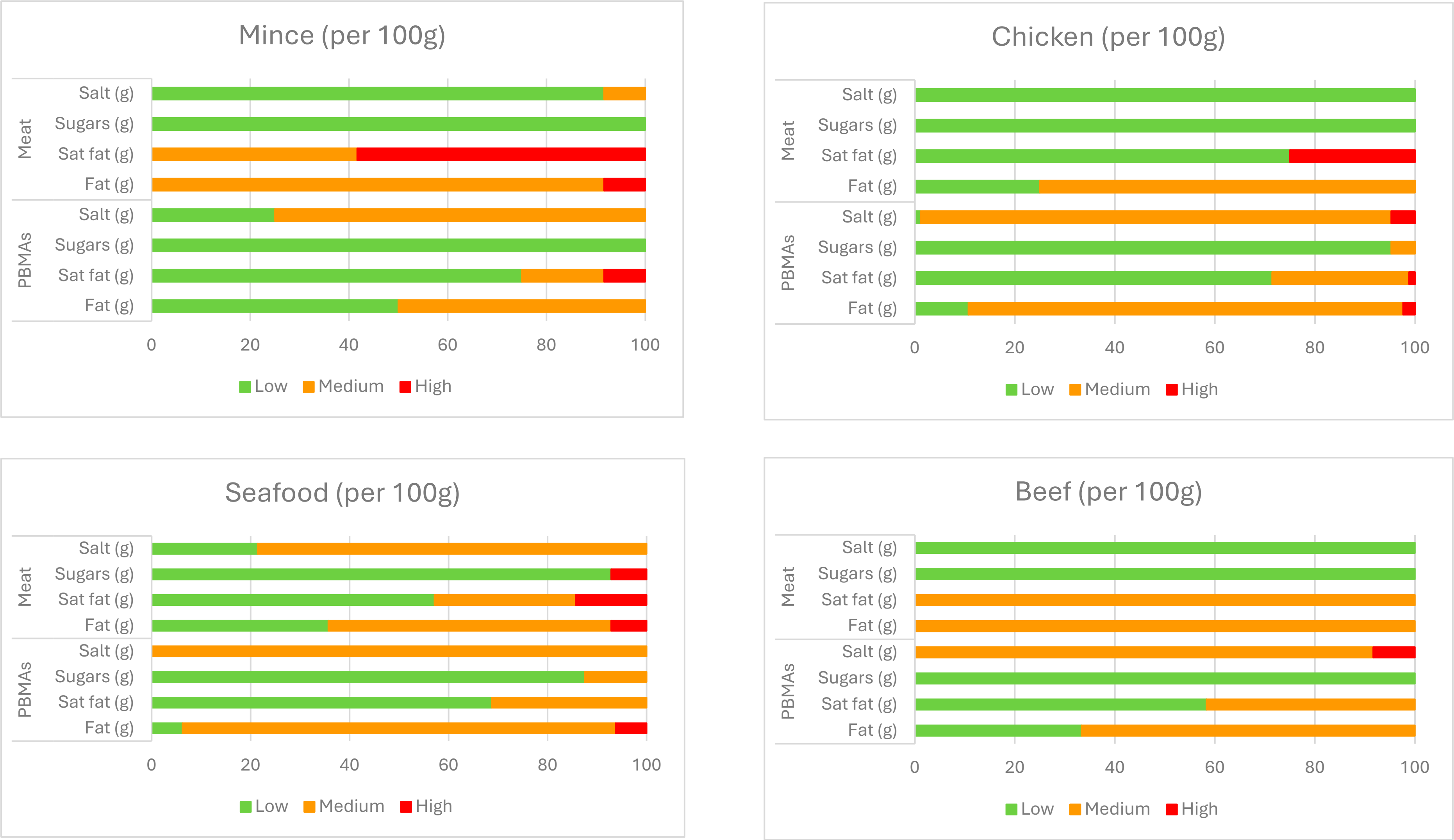

Supplemental Figure S1: Frequency (%) of PB and MB products per category with low, medium and high contents of fat, saturated fat sugars and salt.* *Products were classified as low, medium or high in total fat (≤3g/100g, >3 to ≤17.5g/100g and >17.5g/100g),

saturated fat (≤1.5g/100g, >1.5g to ≤5g/100g and >5g/100g), sugars (≤5g/100g, >5g to ≤22.5g/100g and >22.5g/100g) and salt (≤0.3g/100g, >0.3 to 1.5g/100g and >1.5g/100g) using thresholds from the UK's FoP traffic light labelling system (240).

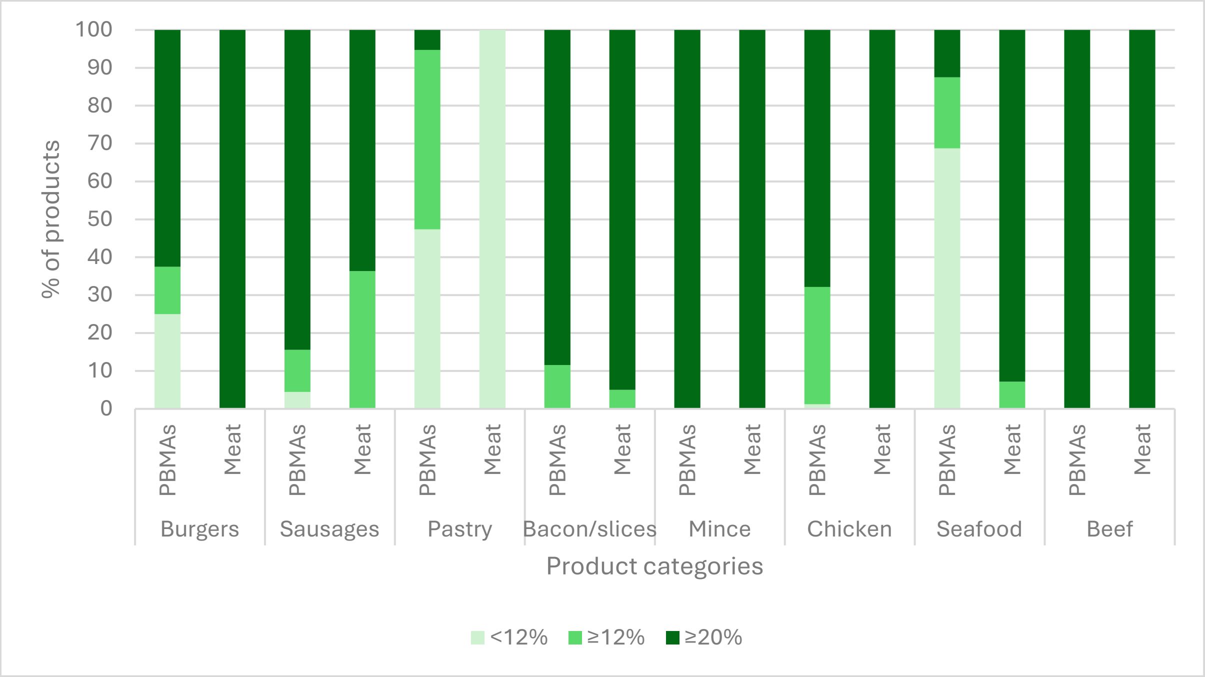

Supplemental Figure S2: Proportion of PBMA and MB products per category with <12%, ≥12% to <20% and ≥20% energy from protein
